# Indirect reciprocity with Bayesian reasoning and biases

**DOI:** 10.1371/journal.pcbi.1011979

**Published:** 2024-04-25

**Authors:** Bryce Morsky, Joshua B. Plotkin, Erol Akçay

**Affiliations:** 1 Department of Biology, University of Pennsylvania, Philadelphia, Pennsylvania, United States of America; 2 Department of Mathematics, Florida State University, Tallahassee, Florida, United States of America; Max Planck Institute for Evolutionary Biology: Max-Planck-Institut fur Evolutionsbiologie, GERMANY

## Abstract

Reputations can foster cooperation by indirect reciprocity: if I am good to you then others will be good to me. But this mechanism for cooperation in one-shot interactions only works when people agree on who is good and who is bad. Errors in actions or assessments can produce disagreements about reputations, which can unravel the positive feedback loop between social standing and pro-social behaviour. Cooperators can end up punished and defectors rewarded. Public reputation systems and empathy are two possible mechanisms to promote agreement about reputations. Here we suggest an alternative: Bayesian reasoning by observers. By taking into account the probabilities of errors in action and observation and their prior beliefs about the prevalence of good people in the population, observers can use Bayesian reasoning to determine whether or not someone is good. To study this scenario, we develop an evolutionary game theoretical model in which players use Bayesian reasoning to assess reputations, either publicly or privately. We explore this model analytically and numerically for five social norms (Scoring, Shunning, Simple Standing, Staying, and Stern Judging). We systematically compare results to the case when agents do not use reasoning in determining reputations. We find that Bayesian reasoning reduces cooperation relative to non-reasoning, except in the case of the Scoring norm. Under Scoring, Bayesian reasoning can promote coexistence of three strategic types. Additionally, we study the effects of optimistic or pessimistic biases in individual beliefs about the degree of cooperation in the population. We find that optimism generally undermines cooperation whereas pessimism can, in some cases, promote cooperation.

## Author summary

Cooperation is an important part of our social lives. However, selfish incentives can undermine it: I need not reciprocate with someone who has cooperated with me. One mechanism to promote cooperation is indirect reciprocity, wherein cooperation is rewarded by good reputations and defection punished by bad ones. This reward is provided indirectly, when other individuals cooperate with those of good reputation and defect against those with a bad reputation. Maintaining accurate reputations is a key part of this mechanism, since mistakes in evaluating others’ behaviours and disagreements about reputations can undermine the feedback loop between reputations and cooperation. Here we develop a model of individual reasoning that can correct for such mistakes and disagreements, and we explore how this impacts the level of cooperation. We find that individual reasoning to determine accurate reputations can often undermine cooperation.

## Introduction

Indirect reciprocity is a well-studied mechanism that can foster cooperation among strangers, even in one-shot interactions [1–3]. Under indirect reciprocity individuals observe others’ behaviours towards third parties, and assign reputations based on a social norm [3, 4]. When individuals then condition their behaviour towards each other based on reputations, for instance rewarding “good” individuals with cooperation and punishing “bad” with defection, this feedback loop can support cooperation. The dynamics of strategies under indirect reciprocity have been thoroughly explored through theoretical models [5], and this phenomenon is empirically observed in both children and adult humans [6–10] as well as non-human animals such as song sparrows [11]. Social norms, which are important in fostering cooperation generally [12, 13], govern the assignment of reputations. Several such norms have been identified as being particularly effective in maintaining cooperation [14].

Though reputations can foster cooperation through indirect reciprocity [15], this only works if reputations are accurate and individuals agree on each others’ reputations. Otherwise, individuals can be wrongly punished or rewarded, leading to a cascade of assigning bad reputations and eventual defection. Importantly, errors in actions or observations can lead to such disagreements between individuals on the reputations of others. Consensus about reputations can be resolved by a public reputation system or by rapid gossip [3, 16, 17]. But when reputations of others are privately held, agreement becomes a difficult problem. Social norms that would otherwise be effective at promoting cooperation under public information, such as Stern Judging and Shunning, fail to establish cooperation under private reputation information [18]. One way to overcome the agreement problem is empathy [19], where observers can evaluate a donor through the eyes of the donor, i.e., using the recipient’s reputation with the donor, which allows private reputations to better coordinate and therefore restore cooperation.

Although errors in actions and in observations play an important role in the theory of indirect reciprocity, theoretical work has assumed individuals are effectively unaware of the possibility of such errors. This means that individuals in these models always take their observations and assessments at face value. In reality, individuals may know that errors in actions or observations do sometimes occur, and they may try to account for these errors when assigning reputations (whether reputations are public or private). When observers know that errors are possible and have beliefs about the expected reputation of others, they can weigh the likelihood of a donor being good given what they have observed. To illustrate, consider an observer who observes a donor defecting against a person they deem good, under a social norm that dictates that such an action is bad. If the observer either knows or believes that there are errors in both the action (the donor intended to cooperate, but somehow failed to do so) and their own observations (the observer may have incorrectly perceived the donor’s action), then they may reasonably be expected to account for this knowledge when forming an opinion of the donor’s reputation. Importantly, this is true regardless of whether reputations are publicly or privately assessed.

In the example above, the observer might consider that there has been an error in action, and the donor actually meant to donate to the recipient, and so should be judged as good. Alternatively, the observer might consider the possibility that they are mistaken in believing that the recipient is good (due to an earlier error in private or public assessment of the reputation), which will change their evaluation of the donor. The observer must weigh these possible scenarios, and assess the donor as good or bad based on their perceptions of the probabilities of errors, their beliefs about prevalence of good individuals, and the social norm. For instance, if an observer believes that the vast majority of people are good and that the probability of an error is relatively high, they would likely give the donor the benefit of the doubt even if on face value they should assess the donor as bad. These considerations—based on Bayesian assignment of reputations—have the potential to change the evolutionary dynamics of cooperation.

Here, we develop a game-theoretical model of indirect reciprocity with Bayesian reasoning about reputation assignment. Players in our model engage in probabilistic reasoning [20]: they balance the probabilities of errors and their beliefs about the frequency of good individuals when forming judgements about others, using Bayes’ Rule. A large literature in cognitive science has proposed that Bayesian processes explain basic aspects of human reasoning and learning [21–30]. Evidence suggests that under some circumstances human cognition may approximate a Bayesian updating process [31], or can be seen as a Bayesian sampler [32]. Bayesian updating of probabilities also can evolve under reasonable evolutionary models [33]. While other ways of reasoning about uncertain evidence exists [34, 35], Bayesian reasoning seems to be a reasonable choice to consider when modeling agents facing uncertainty. Here, we show that when observers using Bayesian reasoning to assess reputations, rather than naively accepting observations as truth, this can dramatically impact the outcome of indirect reciprocity. We consider the cases where reputations are assessed publicly or privately and the effects of biased beliefs about the reputations of others on the dynamics and equilibrium rate of cooperation. Our key finding is that Bayesian reasoning generally reduces rates of cooperation. Under the Scoring norm, however, reasoning can promote cooperation.

## Methods

### Indirect reciprocity

Consider a population of individuals playing a donation game where donors may, at a cost to themselves, provide a benefit to a recipient. We say that those who donate “cooperate” and those that do not donate “defect”. Individuals are chosen at random to meet, one assigned to be the donor and another a recipient. A third individual, the observer, watches their interaction and assigns a reputation to the donor depending on whether or not the donor decides to cooperate and also on the reputation of the recipient (in the eyes of the observer). There is also a chance of errors that could lead to the observer evaluating the donor incorrectly. One type of error is involuntary defection [36]: with probability *e*_1_, a donor intending to cooperate accidentally defects. The other type of error is observational: with probability *e*_2_, an observer observes the wrong action of the donor. The probability that a donor intending to cooperate is correctly observed to be cooperating is thus *ϵ* = (1 − *e*_1_)(1 − *e*_2_) + *e*_1_*e*_2_, and we assume that $1>\epsilon >\frac{1}{2}>e_{2}>0$. Note that, since there is no chance that a donor who intends to defect accidentally cooperates, the probability that a donor intending to defect is correctly observed defecting is 1 − *e*_2_.

We consider an infinite population of individuals engaging in such interactions. There are three strategies: always cooperate (AllC), always defect (AllD), and discriminate (Disc). As donors, AllC and AllD players will always cooperate or defect, respectively, with whomever they are matched. Discriminators, however, discriminate between “good” and “bad” recipients when deciding whether to cooperate. They will cooperate with those they deem good and defect with those they deem bad. The assignment of a reputation to a donor is determined by a set of rules called a social norm. We consider five different social norms most common in the literature: Scoring, Shunning, Simple Standing, Staying, and Stern Judging. The judgments that occur from these norm are summarized in Table 1. For example, under Simple Standing, it is considered good to cooperate with a good recipient, bad to defect against a good recipient, and good regardless of what action is taken towards a bad recipient.

**Table 1:** Assessments of the donor (either *G* or *B* for good or bad) for different social norms.

	*ij*: donor’s action *i* and recipient’s reputation *j*			
Social norm	*CG*	*DG*	*CB*	*DB*
Scoring	*G*	*B*	*G*	*B*
Shunning	*G*	*B*	*B*	*B*
Simple Standing	*G*	*B*	*G*	*G*
Staying	*G*	*B*	—	—
Stern Judging	*G*	*B*	*B*	*G*

The frequencies of the three strategies evolve over time, as players adopt strategies that are more successful than their own. Here we use replicator dynamics [37, 38] to model the changing strategic composition in an infinite population. Let *π*_*i*_ be the expected payoff to type *i*, and *r* > 1 be the benefit to cost ratio of cooperating. The payoffs are thus

$$\pi_{x}=r(x+g_{x}z)-1,\;\pi_{y}=r(x+g_{y}z),\;\pi_{z}=r(x+g_{z}z)-g.$$ (1)

where *x*, *y*, and *z* are the frequencies of AllC, AllD, and Disc players, respectively. *g*_*i*_ is the frequency of *i* individuals with good reputations, and the total number of individuals with good reputations is *g* = *g*_*x*_*x* + *g*_*y*_*y* + *g*_*z*_*z*. These reputations are assessed either publicly or privately. Under public assessment of reputations, an individual is assessed as either good or bad by all individuals and thus there will be agreement on reputations. Under private assessment, on the other hand, individuals assess reputations privately and thus players can disagree on whether an individual is good or bad. As is common in the literature, we assume that reputations equilibrate quickly before strategic frequencies change in the population. The cost to AllC players is 1 and the cost to Discriminators is *g* (since they will only cooperate with those they deem good). The average payoff across all individuals is $\bar{\pi }=\pi_{x}x+\pi_{y}y+\pi_{z}z$. Thus, the replicator equations that govern the dynamics are:

$$\dot{x}=(\pi_{x}-\bar{\pi })x,\;\dot{y}=(\pi_{y}-\bar{\pi })y,\;\dot{z}=(\pi_{z}-\bar{\pi })z.$$ (2)

### Bayesian reasoning

Unlike previous models of indirect reciprocity, we assume observers know the error rates and consider the intentions of donors, when making reputation assessments. Observers know that donors might accidentally defect even if they intend to cooperate, and will attempt to judge them on their *intention* rather than their action. They also know that there is a chance of an assessment (observation) error; and they know the overall frequency of good individuals in the population. The latter information is used in the Bayesian determination of whether the donor is good or not, given an observation. To make a Bayesian assessment, an observer needs to determine the probability that the donor is good given the observed action and knowledge about errors rates the frequency of good individuals in the population. Mathematically, this is:

$$\begin{array}{c} P\left(\text{donor}\;\text{is}\;\text{good}|\text{observation}\right)=\frac{P(\text{observation}|\text{donor}\;\text{is}\;\text{good})P(\text{donor}\;\text{is}\;\text{good})}{P(\text{observation})}. \end{array}$$ (3)

Each time an observer observes an interaction, they assess the donor as good with this probability.

To see how this works, consider a someone who observes a donor cooperating with a good recipient under the Simple Standing norm. Under this norm, it is considered good to contribute to a good recipient. The probability of observing a donor cooperating given that the donor is good and thus intended to give is P(donorcooperateswithagoodrecipient|donorisgood)=ϵ. The probability of assessing a donor as cooperating given that the donor is bad and thus intended to defect is $P\left(\text{donor}\;\text{cooperates}\;\text{with}\;\text{a}\;\text{good}\;\text{recipient}|\text{donor}\;\text{is}\;\text{bad}\right)=e_{2}$. Thus, $P\left(\text{donor}\;\text{cooperates}\;\text{with}\;\text{a}\;\text{good}\;\text{recipient}\right)=\epsilon \hat{g}+e_{2}\left(1-\hat{g}\right)$, i.e. the chance of observing a donor cooperating is the probability of a good individual correctly giving and being assessed as having done so times the frequency of good individuals, and the probability of a bad individual mistakenly being assessed as giving times the frequency of bad individuals. Finally, we have P(donor is good) = $\hat{g}$, the *perceived* frequency of good individuals in the population. As a baseline, we assume that the perceived frequency of good individuals corresponds to the true frequency, corresponding to no biased beliefs, i.e. $\hat{g}=g$. The assessment under Simple Standing for the case where an observer observes a donor cooperating with a good recipient then becomes:

$$\begin{array}{c} P\left(\text{donor}\;\text{is}\;\text{good}|\text{donor}\;\text{cooperates}\;\text{with}\;\text{a}\;\text{good}\;\text{recipient}\right)=\frac{\epsilon \hat{g}}{\epsilon \hat{g}+e_{2}\left(1-\hat{g}\right)}. \end{array}$$ (4)

The assessment rules for the other cases and all other norms are detailed in S1 Text.

We also analyze a generalization of our baseline model to incorporate optimism or pessimism bias in individuals’ beliefs about others’ reputations, as such $\hat{g}\neq g$. Under an optimism bias, $\hat{g}>g$, under a pessimism bias $\hat{g}<g$, and when there is no bias $\hat{g}=g$. Specifically, we model optimism bias by assuming $\hat{g}=\left(1-\lambda \right)g+\lambda$ where 1 > λ > 0 denotes the strength of the bias. Similarly, we assume $\hat{g}=\left(1-\lambda \right)g$ under pessimism bias.

## Results

### Bayesian updating under public assessment of reputations

Here we summarize and discuss the results under public assessment of reputations. Our analysis proceeds as follows: first, we derive the equilibrium reputations in a population composed of a given mixture of AllD, AllC and Disc strategists for Scoring and Shunning, and we prove that reputations converge to a unique stable equilibrium for a given strategic mixture. We use these steady state reputations to calculate the evolutionary dynamics of the three strategic types of players. For the other norms, we prove that there is no strategic equilibrium in the interior of the simplex, and then show convergence of reputations along the AllD-Disc axis and analyze the strategic dynamics. The mathematical details of these results are in S1 Text. Throughout we compare these results to previous literature on indirect reciprocity under public and private assessments without Bayesian reasoning. We denote the results from prior models as the “non-reasoning” case.

**Fig 1:**
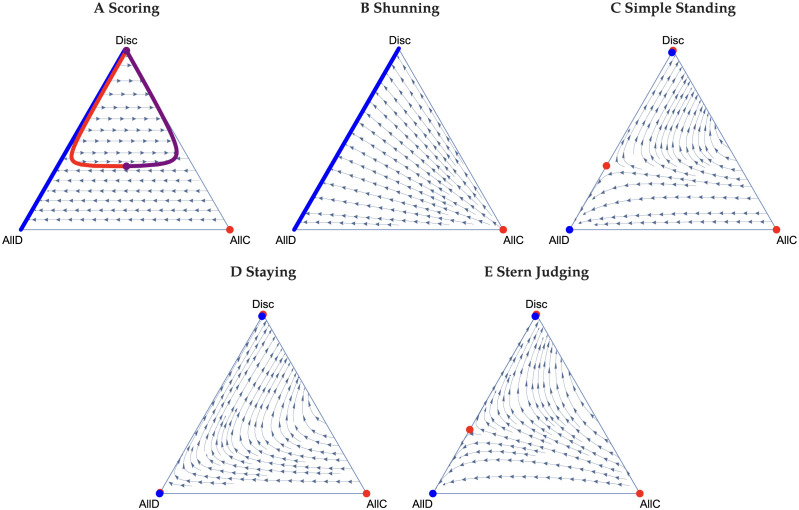
Ternary plots for the leading five norms under public assessment of reputations. Stable, semi-stable, and unstable equilibria are plotted in blue, purple, and red, respectively. Circles are singular equilibria, and lines sets of them. A: For Scoring, if *z* is sufficiently low, then the system can only evolve to the AllD-Disc boundary of the simplex where Discriminators always defect. If *z* is higher, then we observe bistability, which includes a set of equilibria through the interior of the simplex. Note that the red curve representing a set of unstable equilibria is interior to the simplex and only intersects with the blue curve at *z* = 1. The purple curve in turn is semistable since all points along it are attractors except at the end of the curve where Discriminators at their lowest frequency, which is unstable. B: Under Shunning, the AllD-Disc boundary is a stable set of equilibria (note that this also applies to private assessment). C-E: Simple Standing, Staying, and Stern Judging give qualitatively similar results. The system either evolves to AllD or to a position on the AllD-Disc boundary. Note that the stable equilibria on the AllD-Disc boundary are very near *z* = 1. In all figures, the benefit to cost ratio is *r* = 3 and the error rates are *e*_1_ = *e*_2_ = 0.01.

**Fig 2:**
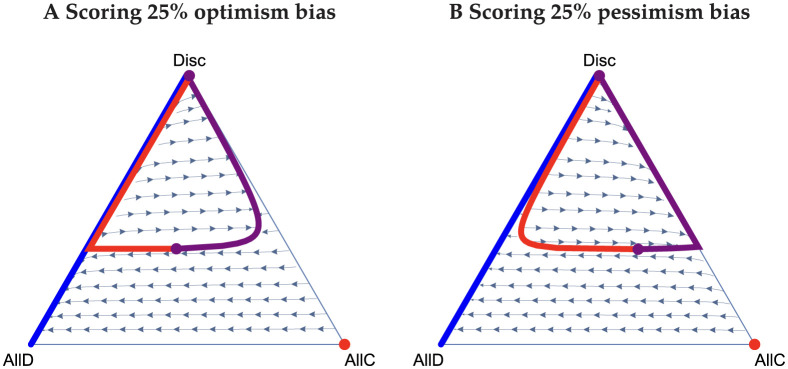
Ternary plots for Scoring under biases, 25% optimism (A) and 25% pessimism (B). Stable, semistable, and unstable equilibria are again plotted in blue, purple, and red, respectively. Circles are singular equilibria, and lines sets of them. These biases have shifted the internal equilibria relative to Fig 1A.

Consider first the Scoring norm, where it is good to cooperate and bad to defect regardless of the recipient’s reputation. In both public and private assessment without reasoning, all players playing AllD is the sole stable equilibrium [18, 39], because errors in action and observation erode the reputation of cooperators and favour AllD. However, with reasoning, we find that cooperation can be sustained. Moreover, and unlike the other social norms, there can be coexistence of all three strategies. (We note that the equations for the frequencies of good individuals for public and private assessment under Scoring are identical, and so all of the following analysis applies to both of them.) If there are sufficiently few Discriminators in the population’s initial state (e.g. the lower portion of the ternary Fig 1A), then the system evolves to pure defection. Without the presence of AllC players, the average reputation *g* goes to zero, and thus Discriminators always defect. Therefore, a mix of AllD and Disc players can coexist at equilibrium. On the other hand, if the frequency of Discriminators is sufficiently high, paths can be attracted to an internal equilibrium supporting positive frequency of all three strategic types. The AllD-Disc boundary is still stable, but now a curve describing a polymorphic population of all three types is semi-stable, e.g. there are regions of phase space where it is attracting and regions where it is repelling. Between these two is a set of unstable equilibria. The semi-stable polymorphic equilibria are in the interior the simplex, which is not observed in any other norms with reasoning. The points on this curve of the semi-stable polymorphic equilibria all support different, positive levels of cooperation. This curve is only semi-stable and not stable, since the end point (where Discriminators are at their nadir) is unstable to perturbations that decrease the frequency of Discriminators. The equilibria are otherwise attracting. We find that the lower the error rate, the larger the region of coexistence of strategies. However, there is a limit on how large it may be, even with no errors. On the other hand, if the error rate is sufficiently large, then the interior equilibria disappear and the only stable equilibrium is the AllD-Disc boundary. The presence of optimism or pessimism bias contorts the semi-stable interior equilibria that arise under the Scoring norm. Optimism bias draws them to the AllD-Disc boundary, and pessimism bias draws them to the AllC-Disc boundary. Fig 2 depicts this phenomena for a 25% optimism bias and a 25% pessimism bias.

Next consider the Shunning norm. Again, public and private assessments lead to qualitatively identical results. Without Bayesian reasoning, Shunning results in either AllD and Disc both being stable equilibria (and the system is thus bistable), or AllD being globally asymptotically stable [18]. With reasoning, we find a stable set of equilibria that excludes cooperation as depicted in Fig 1B. This set exists regardless of the benefit to cost ratio *r*, errors rates, or the presence of bias. It arises because Bayesian reasoning under the Shunning norm drives the reputations of all types to zero. Therefore, Discriminators behave exactly as AllD players and never cooperate.

Finally, we consider Simple Standing, Staying, and Stern Judging together, as they have qualitatively similar dynamics with public assessment of reputations. These three norms can all display bistable dynamics, a monomorphic population of AllD is always stable under all three, but there may be an additional stable equilibrium on the AllD-Disc boundary. Staying has a larger basin of attraction of the cooperative equilibrium (the one with Discriminators) than both Simply Standing and Stern Judging. Fig 1C–1E depict ternary figures for these norms when there is no bias.

**Fig 3:**
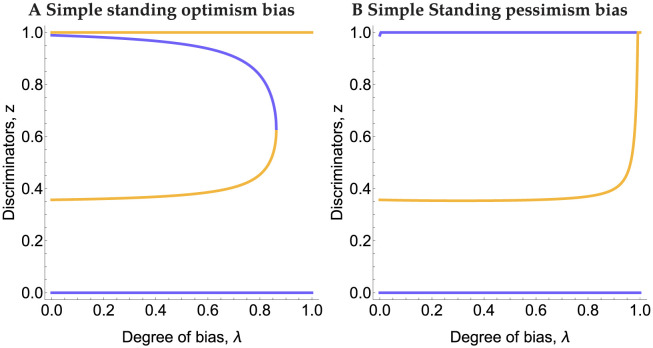
Bifurcation diagrams for optimism (A) and pessimism (B) biases under Simple Standing. Here AllC strategists are excluded and thus 1 − *z* = *y*. Violet curves are stable equilibria and orange curves are unstable.

Bias in the prior beliefs can change the qualitative nature of the equilibria by destroying the equilibrium along the AllD-Disc edge for all three of these norms, and both for optimism and pessimism bias, so long as it is sufficiently large (see S1 Fig for ternary figures with only 25% bias). For optimism bias, the frequency of Discriminators decreases in the edge equilibrium (and therefore the frequency of good players and cooperation) as the degree of bias increases. At a critical bifurcation point, the internal equilibria are annihilated and the sole stable equilibrium is only composed of AllD players. An initial small degree of pessimism bias can benefit Discriminators pushing the equilibrium of only Discriminators. However, for very large negative bias, the basin of attraction to this equilibrium evaporates leaving the monomorphic equilibrium of AllD players as the only stable equilibrium. The bifurcation diagrams in Fig 3 depict these phenomena under Simple Standing. Staying and Stern Judging produce qualitatively similar diagrams (see S2 Fig).

**Fig 4:**
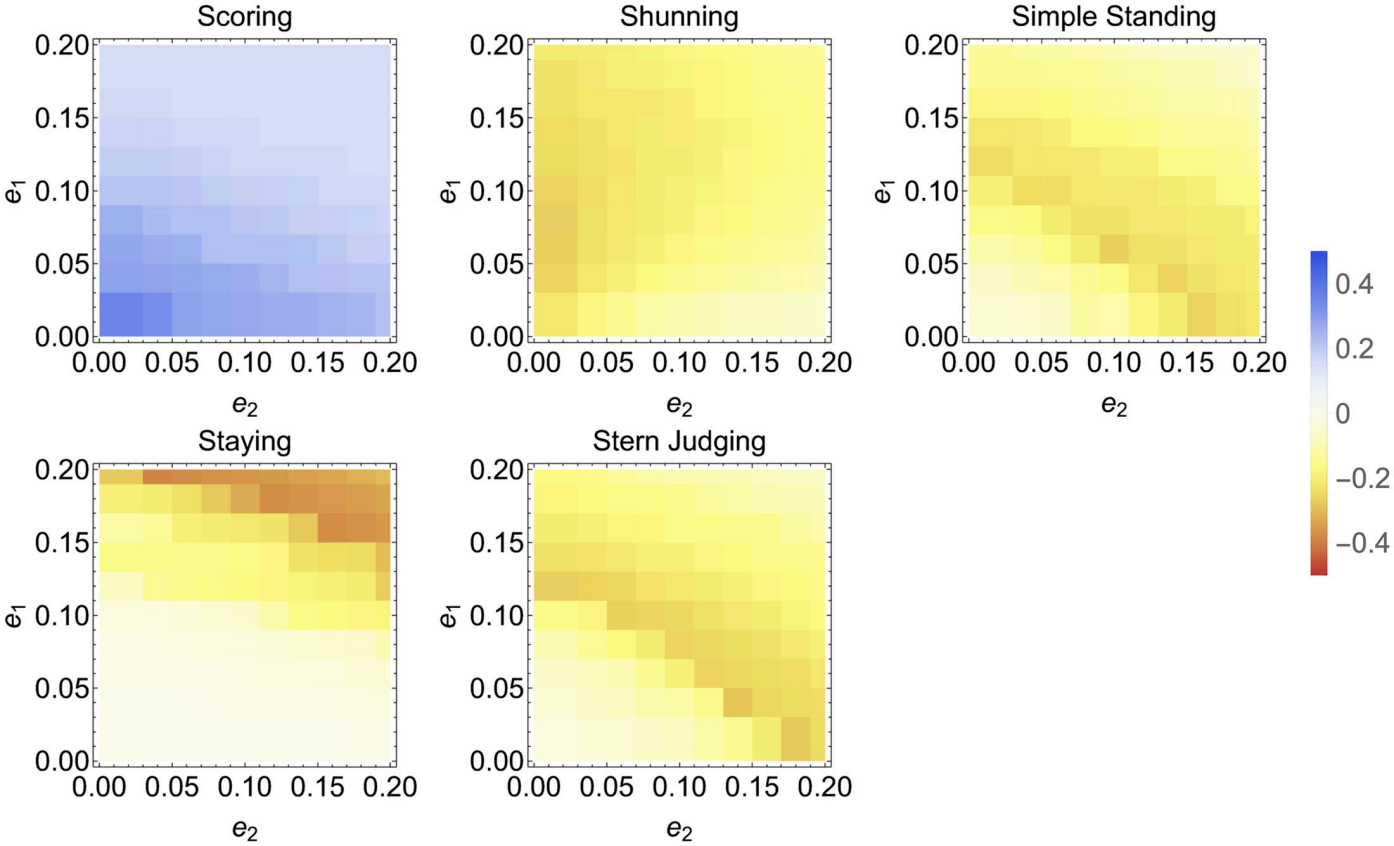
Heat maps of relative cooperation for Scoring, Shunning, Simple Standing, Staying, and Stern Judging, i.e. each cell represents the average amount of cooperation under Bayesian reasoning minus the average amount of cooperation under non-reasoning for different error rates. The average is over initial conditions evenly spread across the simplex and *r* = 3. Note that reasoning generally under-performs compared to non-reasoning except for Scoring.

Thus far, we have fixed the error rates in the figures produced. Here we explore the impact of various error rates on the amount of cooperation at equilibrium for the Bayesian reasoning model compared to the non-reasoning model. These results are plotted in Fig 4. The system is initialized at strategy frequencies evenly spread across the simplex and then numerically solved and averaged. The average frequency of cooperation under non-reasoning is then subtracted from the amount from Bayesian reasoning. Bayesian reasoning generally does worse than non-reasoning: cooperation is lower for Bayesian reasoning than for non-reasoning except for Scoring, which always does better than non-reasoning. Further, Bayesian reasoning generally performs relatively best when error rates are low. We also explored varying errors rates and biases (results in S3 Fig).

### Private assessment of reputations

**Fig 5:**
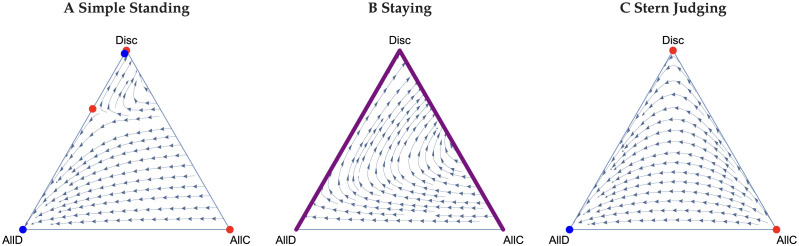
Ternary plots for Simple Standing, Staying, and Stern Judging under private assessment. Stable and unstable equilibria are plotted in blue and red, respectively. Circles are singular equilibria and lines sets of them. A: Simple Standing gives qualitatively similar results to Public assessment. The system either evolves to AllD or a point on the interior of the AllD-Disc boundary. For this panel, *e*_1_ = *e*_2_ = 0.05 so that the plotting of the equilibria are clear. For *e*_1_ = *e*_2_ = 0.01, the interior stable equilibrium is nearly on the Disc vertex. B: Under Staying, the AllC-Disc and AllD-Disc boundaries are sets of equilibria. Further, at z* = 1, any reputation is an equilibria. Thus, the trajectory towards z* = 1 will determine reputations at it. C: Under Stern Judging, AllD is globally asymptotically stable. *r* = 3 for all figures, and *e*_1_ = *e*_2_ = 0.01 for panels B and C.

Here we consider private assessment of reputations. Since Scoring and Shunning norms result in the same behaviour for public and private assessment, we focus on Simple Standing, Staying, and Stern Judging. Under Simple Standing, in the absence of any bias, cooperation can be maintained. However, AllC strategists cannot be part of an equilibrium. Therefore, all equilibria are along the AllD-Disc boundary. We also find that the monomorphic AllD equilibrium (y* = 1) is always stable and the monomorphic Disc equilibrium (z* = 1) is always unstable. Between these two equilibria there may be zero, one, or two equilibria depending on the benefit to cost ratio *r*, and error rates *e*_1_ and *e*_2_ (see S1 Text for details of these conditions). For parameters where there is no equilibrium along the Disc-AllD line, AllD (y* = 1) is globally stable. If there is one equilibrium containing a mixture of AllD and Disc, it is semi-stable along the AllD-Disc boundary, i.e. it is stable coming from the direction of AllD, and unstable from the direction of Disc. In the case where there are two equilibria, then the one closest to z* = 1 is stable, while the other is not, and the dynamics of the system converge to either to the monomorphic AllD or the stable mixture of AllD and Disc. The latter equilibrium can maintain a high level of cooperation as it can mostly consist of Discriminators. Fig 5A depicts this case for *r* = 3 and *e*_1_ = *e*_2_ = 0.01. Note that in the figure, the stable mixture is mostly Discriminators so that it is very close to the corner with all Discriminators (which itself is an unstable equilibrium).

**Fig 6:**
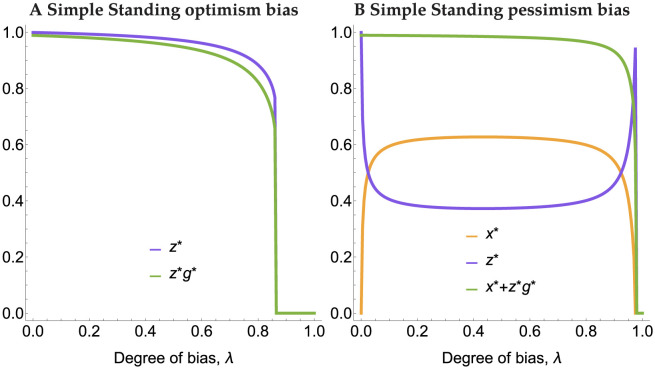
Equilibria x* (orange), z* (violet), and the equilibrium degree of cooperation x* + g*z* (green) for varying degrees of optimism and pessimism biases under Simple Standing.

Biases in prior beliefs about reputations of others can have a dramatic impact on the dynamics of the strategies under Simple Standing. Fig 6 depicts the most cooperative equilibrium (corresponding to the AllD-Disc mixture described above without bias), and the amount of intended cooperation at equilibrium (i.e. x* + g*z*). Fig 6A shows that increasing optimism bias decreases the frequency of Disc players at equilibrium and reduces the total amount of cooperation. This happens because optimism bias causes Discriminators to evaluate more individuals as good and cooperate with them when they should not, which favours AllD strategists. This effect is relatively mild except for very high degree of optimism bias, where the gullibility of Discriminators drives them extinct and the population consists entirely of AllD strategists. Negative bias (Fig 6B) displays a qualitatively different pattern: here, even for small amount of bias, discriminator frequency at equilibrium goes down significantly, but now they are replaced by AllC strategists. This happens because pessimism bias causes Discriminators to defect against individuals judged as good by others, which makes them subject to punishment by other Discriminators. AllC strategists, who always cooperate, do not suffer from this punishment and can increase in frequency. Strikingly, when pessimism bias increases further, this effect is reversed: AllC strategists start getting punished as much as Discriminators, and lose their advantage, which causes Discriminators to increase in frequency at high pessimism biases. This exchange of Disc and AllC strategists causes little change in the overall level of cooperation for most degrees of pessimism bias, except for when pessimism bias is so high that Discriminators regard everyone to have bad reputations and defect against them. At this point (the far right hand side of Fig 6B), Discriminators get replaced by AllD strategists.

Fig 5B depicts a ternary plot for Staying. The AllC-Disc and AllD-Disc boundaries both form sets of equilibria since g* = 1 and g* = 0 on each, respectively. y* = 1 is therefore semi-stable, since perturbations from it along the AllD-Disc boundary are neutral and trajectories with sufficiently low *z* converge to it. Perturbations to z* = 1 may also occur neutrally along the AllD-Disc boundary in addition to along the AllC-Disc boundary. z* = 1 is attracting in the interior of strategy space. Further, all reputations are equilibria at z* = 1. Therefore, g* at z* = 1 will depend on the trajectory approaching it. Both this result for Staying and that for Simple Standing are in contrast to non-reasoning models of private assessment, where there is a polymorphic stable equilibrium of Discriminators and AllC players [18], and public assessment, where z* = 1 is stable [39].

Finally, Bayesian reasoning makes no impact on Stern Judging when there is no bias. Like the non-Bayesian case under private assessment [18], the equilibrium reputations for all strategies are always $\frac{1}{2}$. Therefore, *π*_*y*_ > *π*_*z*_ > *π*_*x*_, which results in all players playing AllD as depicted in Fig 5C. Even moderate bias has no discernible effect on outcomes (see S4 Fig for ternary plots of Stern Judging along with Staying and Simple Standing with λ = 0.25).

**Fig 7:**
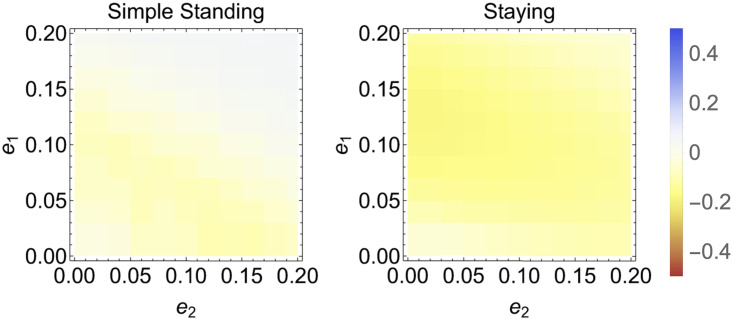
Heat maps of relative cooperation for Simple Standing and Staying, i.e. cooperation under Bayesian reasoning minus cooperation under non-reasoning. Results are averaged over initial conditions evenly spread across the simplex, with *r* = 3.

Fig 7 shows heat maps for varying error rates under private assessment for Simple Standing and Staying (Scoring is not presented as the results are identical to public assessment of reputations, and Shunning and Stern Judging are not presented because they always lead to defection). Bayesian reasoning generally under-performs compared to non-reasoning, although this effect is small and it is lessened when error rates are low. One exception, however, is that for high error rates, Simple Standing has approximately the same degree of cooperation as or more than non-reasoning. Since these results show a marginal difference between Bayesian reasoning and non-reasoning and since they are generated from discretizing the space and numerical simulations, we cannot conclude that Bayesian reasoning can significantly promote cooperation. Rather, error rates have a marginal impact on the relative outcomes particularly with respect to Simple Standing. We also explored varying errors rates and biases (results in S5 Fig).

## Discussion

Indirect reciprocity is one of the main mechanisms that can sustain cooperation among strangers [3]. But this mechanism requires keeping track of reputations in the population, and it also requires some degree of mutual agreement about reputations in the population. In particular, Discriminators must agree on who the “bad” people are so that they can effectively punish them while rewarding those who are “good.” When evaluations of others are privately held, this coordination can break down, and with it cooperation maintained by indirect reciprocity. Public institutions that decide and promulgate reputations centrally [16] or individuals putting themselves in others’ shoes, i.e., empathy [19], can solve this problem, however even in publicly shared reputations, individuals still have to contend with errors of action and observation. There is a large body of evidence suggesting that humans, when they need to make decisions or learn in the presence of errors and noise, employ Bayesian reasoning to take into account the sources of uncertainty [24, 25, 40]. We have shown that Bayesian reasoning about two sources of error, in action and perception, can substantially alter social behaviour in the context of reputations.

Bayesian reasoning about the sources of error can sometimes promote cooperation that would otherwise be suppressed. The most striking example of this happens with Scoring, which is an attractive social norm since it is first order (individuals’ reputations only depend on their own behaviour, not the recipients) and thus presents low cognitive and informational requirements. In most theoretical treatments, however, Scoring is plagued by the fact that, in the presence of AllC, errors gradually erode reputations and, subsequently, cooperation [5, 39, 41]. Particularly, inaccurate information can prevent the evolution of social norms and cooperation [41]. Bayesian reasoning can counteract this erosion, because it allows individuals to overlook errors provided they have sufficiently high prior expectations for good reputations in the population. In this way, individuals can avoid mistakenly assigning bad reputations to cooperators and Discriminators and maintain the relative reward these types enjoy. This process can maintain a mixed equilibrium with all strategic types. Specifically, AllD can also exist in these equilibria because they are given a measure of relief as defection is sometimes ascribed to errors. Notably, however, this only works if the population starts from a sufficient fraction of Discriminators; otherwise the AllD-Disc boundary is the only attractive equilibrium. [42] also found attractive interior equilibria in a model that has no observation error but potentially incomplete observation of past history. Such interior stable sets of equilibria disappear when there are both observation and action errors [18], yet they return in a different form when Bayesian reasoning is introduced.

Bayesian reasoning under public information has more complicated effects for second-order norms, where the assessment of a donor’s action depends on the recipient’s reputation. Under the very strict Shunning norm (where interacting with bad individuals at all is bad, regardless of outcome) for example, Bayesian reasoning precludes a cooperative equilibrium that exists with public reputations and no reasoning [18, 39]. That equilibrium exists in the non-reasoning case because errors about recipient reputation ameliorates the erosion of reputation for Disc and AllC strategists that interact with bad individuals, so that a reputational equilibrium with positive probably of being good is possible for these types. Bayesian reasoning removes this refuge and all reputations evolve to bad. The other three norms we have studied (Simple Standing, Staying, and Stern Judging) show relatively smaller difference with the non-reasoning case, all showing bistability with one AllD and one cooperative equilibrium. However under Bayesian reasoning, the cooperative equilibrium is a mixture of Disc and AllD, instead of Disc and AllC, which all things being equal results in lower cooperation. And so reasoning can be a double=edged sword when it comes to maintaining cooperation, depending on the social norm of judgement.

Considering probabilistic reasoning about reputations also allows us to model the potential for biased beliefs about others. Classic results in social psychology show that humans attend to and retain negative social information or signals more readily [43, 44]. These biases might lead actors to underestimate the frequency of good individuals in their prior beliefs (pessimism bias). On the other hand, other well-established biases in perception and decision-making such as optimism bias [45] or ego-centric bias [46] can induce actors to overestimate the frequency of good individuals. While the social psychology research on biases in social perception and inference is vast, to our knowledge its effect on the dynamics of indirect reciprocity had not been studied.

We have shown that biases in beliefs of others can sometimes help sustain cooperators (though not necessarily cooperation). And yet excessive biases tend to unravel cooperative behaviour, resulting in negative reputations for everyone and defection rampant. In both cases, the effects are dependent on the norm: with Scoring, for example, optimism bias (thinking others are good more often than they really are) can increase the basin of attraction of the internal equilibrium curve with cooperation, but pessimism bias shrinks it. With Simple Standing (under public reputations), however, optimism bias lowers the degree of cooperation as it makes Discriminators susceptible to cooperating with AllD strategists. Pessimism bias however, can help Discriminators. Both positive and negative bias, however, destroys cooperative equilibria when it is extreme, as Discriminators with highly biased beliefs make uninformative judgments.

There are several limitations and assumptions we have made that may provide interesting avenues for future research. For one, we have assumed an infinite population and no stochasticity. We expect that a model featuring stochasticity and a large but finite population would behave similarly to our model. Except there may be key differences with respect to the continuous sets of equilibria. A finite population experiencing stochasticity could move along these sets of equilibria, which could act as a bridge from one region of phase space to another. We have also assumed no cognitive constraints or complexity costs [47] on individuals, which is an important factor [48]. Previous research on indirect reciprocity that has explored complexity costs for Discriminators finds that such costs can undermine cooperation [42, 47, 49]. Were we to impose such costs here, they may exacerbate the failures of Bayesian reasoning to promote cooperation since Discriminators would earn lower payoffs. On the other hand, this effect may promote cooperators relative to Discriminators, which in turn could promote overall cooperation. It is also of note that Bayesian reasoning tends to be beneficial under the simplest norm and thus arguably the norm with the lowest complexity cost (since observers need not factor in the reputation of the recipient). Whether or not Bayesian reasoning might be advantageous with a complexity cost is thus an open question. Though cognitive costs for reciprocity may not be high [50], they may be substantial for the type of reasoning employed here and sufficient enough to impact the qualitative behaviour of the system. In addition to these points, the ability to use Bayesian reasoning can vary between individuals [51, 52], whereas we have assumed all agents reason in the same way.

Another simplifying assumption is that even with private assessments of individual reputations, beliefs about the error rates and the overall frequency of good individuals are homogeneous and, with respect to the error rates, accurate. Under private assessment of reputations, individuals could obtain such accurate and homogeneous information through their own and others’ past personal experience and repeated interactions (or, under public reputation, through institutions). But what happens if such learning is not feasible or happens only slowly remains an open question. Under the norms where there is bistability, if a population initially has a high belief in the frequency of good individuals, that might be enough to bootstrap cooperative equilibria [53]. For error rates, individuals who intend to cooperate may know the rate at which they fail to cooperate, *e*_1_, through their own involuntary failures to cooperate, which could be transmitted via gossip to others. Similarly, the observational error, *e*_2_, may be learned through gossip when individuals learn they have been inaccurately assessed. At the same time, another well-documented cognitive bias, overconfidence [54], might lead to error rates to be underestimated.

Rationality and probabilistic reasoning have an important role in economic theory [55]. And, it has been argued that humans engage in Bayesian rationality to reason about uncertainty [56]. Our model of indirect reciprocity can be interpreted in this sense. However, surprisingly, reasoning in this matter is not always effective in promoting cooperation, in our analysis. For cooperation to be fostered, AllD players must receive relatively low payoffs, which is not the same as uncovering the true frequencies of good individuals and agreeing on reputations.

## Supporting information

S1 Text Analytical results. (PDF)

S1 Fig Ternary plots for Simple Standing, Staying, and Stern Judging under public assessment of reputations with bias λ = 0.25. The benefit to cost ratio is *r* = 3 and the error rates are *e*_1_ = *e*_2_ = 0.01. The results are not qualitatively different from when there is no bias.
(PDF)

S2 Fig Bifurcation diagrams for optimism and pessimism biases under public assessment of reputations and for Staying and Stern Judging. Here AllC strategists are excluded and thus 1 − *z* = *y*. Violet curves are stable equilibria and orange curves are unstable. The results are qualitatively similar to that of Simple Standing in the main text.
(PDF)

S3 Fig Heat maps of relative cooperation for Scoring, Simple Standing, Staying, and Stern Judging under public assessment of reputations and for different error rates and degrees of bias λ. Each cell represents the average amount of cooperation under Bayesian reasoning minus the average amount of cooperation under non-reasoning. The average is over initial conditions evenly spread across the simplex and *r* = 3. We observe a slight synergy between the error rates and bias for Scoring under optimism bias, Simple Standing and Stern Judging under pessimism bias, and public assessment of reputations: Bayesian reasoning generally has relatively lower cooperation when biases are large and errors low.
(PDF)

S4 Fig Ternary plots for Simple Standing, Staying, and Stern Judging under private assessment of reputations with bias λ = 0.25. The benefit to cost ratio is *r* = 3 and the error rates are *e*_1_ = *e*_2_ = 0.01. Bias has no great impact on the qualitative outcome, except that private Staying no longer has unstable equilibria on two boundaries. For optimism bias and Staying, the plot is qualitatively similar to public assessment of reputations. Negative bias results in a stable equilibrium along the AllC-Disc boundary.
(PDF)

S5 Fig Heat maps of relative cooperation for Scoring, Simple Standing, Staying, and Stern Judging under private assessment of reputations and for different error rates and degrees of bias λ. Each cell represents the average amount of cooperation under Bayesian reasoning minus the average amount of cooperation under non-reasoning. The average is over initial conditions evenly spread across the simplex and *r* = 3.
(PDF)
