# Supplementary material for: Indirect reciprocity with Bayesian reasoning and biases
Source: PLoS Comput Biol. 2024 Apr 25;20(4):e1011979. doi: 10.1371/journal.pcbi.1011979 (PMC11045068; doi:10.1371/journal.pcbi.1011979)
Supplement: S1 Text — (PDF) [file pcbi.1011979.s001.pdf]

Supplementary Information for

Indirect reciprocity with Bayesian reasoning and biases

Bryce Morsky [*1,2], Joshua B. Plotkin [†1], and Erol Akçay [‡1]

[1]Department of Biology, University of Pennsylvania, Philadelphia, PA, USA

[2]Department of Mathematics, Florida State University, Tallahassee, FL, USA

March 22, 2024

Here we detail the analysis of the replicator-dynamic model under the different norms. For notational simplicity, we let $e = e_2$ be the assessment/observational error, and $P_{ij} = \mathbb{P}(G|ij)$ be the probability that the donor is good given that the observer believes they did action i (cooperate C or defect D) and the recipient's reputation with the observer is j (good G or bad B). We also define Q_{ij} as the probability that a donor is good where their *intended* action is i (cooperate C or defect D), and the recipient's reputation with the observer is j (good G or bad B). Therefore, we have:

$$\begin{aligned} Q_{CG} &= \epsilon P_{CG} + (1-\epsilon)P_{DG}, \qquad Q_{DG} = (1-e)P_{DG} + eP_{CG}, \\ Q_{CB} &= \epsilon P_{CB} + (1-\epsilon)P_{DB}, \qquad Q_{DB} = (1-e)P_{DB} + eP_{CB}. \end{aligned} \tag{1}$$

As is common in this modelling literature [1] we analyze strategy dynamics and reputation dynamics by separation of timescales – letting reputations equilibrate quickly, before strategy frequencies change.

The analysis of reputation dynamics requires the probabilities g_i^+ and g_i^-, which are the probabilities of the reputation of type-i individuals increasing and decreasing, respectively. For private assessment, we also require g_{i2} and $g_2 = xg_{x2} + yg_{y2} + zg_{z2}$ where g_{i2} is the probability that two Discriminators agree that an i player is good. g_{i2}^+ and g_{i2}^- are the probabilities that g_{i2} increases or decreases, respectively. Assuming continuous dynamics, we can model reputation dynamics as a set of ordinary differential equations on a fast timescale relative to strategic dynamics. For public assessment these ODEs are $\dot{g}_i = g_i^+ - g_i^-$ for $i = x, y, z$. Private assessment has these equations along with $\dot{g}_{i2} = g_{i2}^+ - g_{i2}^-$ for $i = x, y, z$. Note that when we combine the replicator and reputation dynamics, we must take into account that the reputation dynamics converge more quickly than the replicator dynamics. Thus, when combined, we have the equations $\dot{g}_i = \tau(g_i^+ - g_i^-)$ and $\dot{g}_{i2} = \tau(g_{i2}^+ - g_{i2}^-)$ for $\tau \gg 1$ along with the replicator equation (Equation

*bmorsky@fsu.edu
†jplotkin@sas.upenn.edu
‡eakcay@sas.upenn.edu

3 in the main text).

Below, we present analytical results for the reputation dynamics and the resulting replicator dynamics for the strategies, proving a number of results for each norm. The appendix is organized by the different norms; under each norm, we consider the cases of public and private assessment of reputations, as well as optimism and pessimism bias.

1 Scoring

Since the evaluation of donors under Scoring does not depend upon the reputation of the recipient, the probabilities that the donor is good given the observers' beliefs about their actions and the recipients' reputations with the observers are:

$$P_{CG} = P_{CB} = \frac{\epsilon \hat{g}}{\epsilon \hat{g} + e(1-\hat{g})}, \qquad P_{DG} = P_{DB} = \frac{(1-\epsilon)\hat{g}}{(1-\epsilon)\hat{g} + (1-e)(1-\hat{g})}. \tag{2}$$

Thus, $Q_{CG} = Q_{CB}$ and $Q_{DG} = Q_{DB}$.

Consider first the public assessment of reputations. Since the reputation of the recipient is not a factor in assessments, we can simply focus on the intention of the donor. A donor is good if and only if they give. Therefore, $g_x = Q_{CG}$, $g_y = Q_{DG}$, and $g_z = Q_{CG}g + Q_{DG}(1-g)$. Now, under private assessment, the probabilities of reputation changes are:

$$\begin{aligned}
g_x^+ &= (1-g_x)Q_{CG}, & g_x^- &= g_x(1-Q_{CG}), \\
g_y^+ &= (1-g_y)Q_{DG}, & g_y^- &= g_y(1-Q_{DG}), \\
g_z^+ &= (1-g_z)(Q_{CG}g + Q_{DG}(1-g)), & g_z^- &= g_z((1-Q_{CG})g + (1-Q_{DG})(1-g)).
\end{aligned} \tag{3}$$

At the steady state $g_i^+ = g_i^-$, we have $g_x = Q_{CG}$, $g_y = Q_{DG}$, and $g_z = Q_{CG}g + Q_{DG}(1-g)$, which is identical to the case of public assessment. Note that if there is no bias, $g_z = Q_{CG}g + Q_{DG}(1-g) = g$. The following analyses thus apply to both public and private assessment under Scoring.

Theorem 1.1. *Assume that there is no bias ($\hat{g} = g$). Then, at reputation equilibrium, $g^* = x/(x+y)$, and thus $\dot{z} = 0$ everywhere.*

Proof. Consider first the interior of the simplex. Plugging in the equilibrium reputations $g_x = Q_{CG}$, $g_y = Q_{DG}$, and $g_z = Q_{CG}g + Q_{DG}(1-g)$ into $g = xg_x + yg_y + (1-x-y)g_z$ and simplifying gives us:

$$\frac{(\epsilon - e)^2 g(1-g)((x+y)g - x)}{(\epsilon g + e(1-g))((1-\epsilon)g + (1-e)(1-g))} = 0. \tag{4}$$

The denominator is always positive, so the solutions are $g^* = 0$, $x/(x+y)$, and 1. $g^* = 0 \implies g_x^* = g_y^* = g_z^* = 0$ and

$g^* = 1 \implies g_x^* = g_y^* = g_z^* = 1$. $g^* = x/(x+y)$ implies:

$$g_x^* = \frac{x(\epsilon(1-\epsilon)x + (e + \epsilon(\epsilon - e))y) + e(1-2\epsilon)y)}{((1-\epsilon)x + (1-e)y)(\epsilon x + ey)}, \quad g_y^* = \frac{x(\epsilon(1-\epsilon)x + e(1-e)y)}{((1-\epsilon)x + (1-e)y)(\epsilon x + ey)}, \quad g_z^* = \frac{x}{x+y}. \tag{5}$$

We analyze the stability of the change in reputations $\dot{g}_i = g_i^+ - g_i^-$. Recalling that $g_z = g$, the Jacobian of this reputation system is:

$$J = \begin{pmatrix} \frac{dQ_{CG}}{dg_x} - 1 & \frac{dQ_{CG}}{dg_y} & \frac{dQ_{CG}}{dg_z} \\ \frac{dQ_{DG}}{dg_x} & \frac{dQ_{DG}}{dg_y} - 1 & \frac{dQ_{DG}}{dg_z} \\ x & y & z-1 \end{pmatrix}. \tag{6}$$

Analyzing the system in the interior of the simplex, the eigenvalues λ_i of J at the equilibria g^* are:

$$\begin{aligned} g^* = 0 &\implies \lambda_1 = \lambda_2 = -1, \lambda_3 = \frac{(\epsilon - e)^2 x}{e(1-e)} > 0, \\ g^* = \frac{x}{x+y} &\implies \lambda_1 = \lambda_2 = -1, \lambda_3 = \frac{-(\epsilon - e)^2 xy(x+y)}{((1-\epsilon)x + (1-e)y)(\epsilon x + ey)} < 0, \\ g^* = 1 &\implies \lambda_1 = \lambda_2 = -1, \lambda_3 = \frac{(\epsilon - e)^2 y}{\epsilon(1-\epsilon)} > 0. \end{aligned} \tag{7}$$

Since there are positive eigenvalues for $g^* = 0$ and $g^* = 1$, they are unstable. All of the eigenvalues for $g^* = x/(x+y)$ are negative, and thus it is the unique stable equilibrium. Therefore, reputations will equilibrate at $g^* = x/(x+y)$. Plugging this value into the replicator equation (i.e. the equation for the strategic dynamics, Equation 3) for z gives us:

$$\begin{aligned} \dot{z} &= (\pi_z - \bar{\pi})z = (\pi_z(1-z) - \pi_x x - \pi_y y)z \\ &= ((r(x + g_z^* z) - g)(x+y) - (r(x + g_x^* z) - 1)x - (r(x + g_y^* z))y)z \\ &= (x - g^*(x+y) + r(g_z^*(x+y) - g_x^* x - g_y^* y)z)z \\ &= (x - g^*(x+y) + r((Q_{CG} g^* + Q_{DG}(1-g^*))(x+y) - Q_{CG} x - Q_{DG} y)z)z \\ &= (x - g^*(x+y) - r(Q_{CG}(x - g^*(x+y)) - Q_{DG}(x + y - g^*(x+y) - y))z)z \\ &= (x - g^*(x+y) - (x - g^*(x+y))r(Q_{CG} - Q_{DG})z)z \\ &= (x - g^*(x+y))(1 - r(Q_{CG} - Q_{DG})z)z = 0, \end{aligned} \tag{8}$$

since $g^* = x/(x+y)$. We can conduct a similar analysis on the boundaries of the simplex. On the AllC-AllD boundary, we have the typical Prisoner's Dilemma, where AllD is stable. On the AllC-Disc boundary, subbing in $g_x = Q_{CG}$ and $g_z = Q_{CG} g + Q_{DG}(1-g)$ into $g = x g_x + (1-x) g_z$ gives us:

$$\frac{(\epsilon - e)^2 x g (1-g)^2}{(\epsilon g + e(1-g))((1-\epsilon)g + (1-e)(1-g))} = 0 \tag{9}$$

with solutions $g^* = 0$ and 1. The eigenvalues at $g^* = 0$ are:

$$\lambda_1 = -1, \lambda_2 = \frac{(\epsilon - e)^2 x}{e(1-e)} > 0, \tag{10}$$

and thus it is unstable. Since the system is two dimensional, the other equilibrium $g^* = 1$ must be stable. Further, at $g^* = 1$, Discriminators behave identically to AllC players, and thus $\dot{z} = 0$.

Finally, on the AllD-Disc boundary, subbing in $g_y = Q_{DG}$ and $g_z = Q_{CG} g + Q_{DG}(1-g)$ into $g = y g_y + (1-y) g_z$ gives us:

$$\frac{(\epsilon - e)^2 y g^2 (1-g)}{(\epsilon g + e(1-g))((1-\epsilon)g + (1-e)(1-g))} = 0 \tag{11}$$

the only solutions are $g^* = 0$ and 1. The eigenvalues at $g^* = 1$ are:

$$\lambda_1 = -1, \lambda_2 = \frac{(\epsilon - e)^2 y}{\epsilon(1-\epsilon)} > 0, \tag{12}$$

and thus it is unstable. Since the system is two dimensional, the other equilibrium $g^* = 0$ must be stable. Further, at $g^* = 0$, Discriminators behave identical to AllD players, and thus $\dot{z} = 0$. □

Theorem 1.2. *Assume that there is no bias ($\hat{g} = g$). Then, there is an internal set of equilibria that satisfies $r(Q_{CG} - Q_{DG})z^* = 1$. This set can be divided into continuous semi-stable and unstable subsets. Further, as the error rates increase, z^* increases.*

Proof. $\pi_x = \pi_y = \pi_z \implies r(Q_{CG} - Q_{DG})z^* = 1$ for the internal equilibria. Since $\dot{z} = 0$ by Theorem 1.1, $\dot{x} = -\dot{y}$ and thus we can only concern ourselves with the dynamics of x. At $g^* = x/(x+y)$,

$$\begin{aligned}\dot{x} &= (r(Q_{CG} - Q_{DG})z^* - 1)\frac{xy}{x+y} \\ &= \left(\frac{(\epsilon-e)^2(1-rz^*)x^2 + (\epsilon-e)((\epsilon-e)rz^* - 1 + 2e)(1-z^*)x - e(1-e)(1-z^*)^2}{((1-\epsilon)x + (1-e)y)(\epsilon x + ey)}\right)\frac{xy}{x+y}.\end{aligned} \tag{13}$$

As $x \to 0$ or $x \to 1$, $r(Q_{CG} - Q_{DG})z^* - 1 \to -1$. Sign changes are determined by the numerator within the bracket, which is a concave down quadratic function with respect to x where there are internal equilibria. Since, $1 > Q_{CG} - Q_{DG} > 0$ and $r(Q_{CG} - Q_{DG})z^* = 1$ implies that $rz^* > 1$, and thus the coefficient of the x^2 term is $(\epsilon - e)^2(1 - rz^*) < 0$. Thus, the equilibrium closest to $x = 1$, the "right hand" set, is stable as $\dot{x}$ is positive and negative with x perturbed lower and higher, respectively. Note, however, that this right hand set is unstable at its lowest z value, since below this point the strategies evolve to the AllD-Disc boundary. Hence, the right hand set is semi-stable. The other equilibrium, the "left hand" set, is unstable, since $\dot{x}$ is negative and positive with x perturbed lower and higher, respectively.

Note that in the interior of the simplex we have:

$$
\begin{aligned}
Q_{CG} - Q_{DG} &= \frac{(\epsilon - e)^2 g(1-g)}{(\epsilon g + e(1-g))((1-\epsilon)g + (1-e)(1-g))} \geq 0, \\
\frac{\partial(Q_{CG} - Q_{DG})}{\partial e_1} &= \frac{-(1-e_1)(1-2e_2)^2 g(1-g)(2e_2(1-e_2) + (1-e_1)(1-2e_2)^2 g)}{((\epsilon g + e(1-g))((1-\epsilon)g + (1-e)(1-g)))^2} < 0, \\
\frac{\partial(Q_{CG} - Q_{DG})}{\partial e_2} &= \frac{-(1-e_1)^2(1-2e_2)g(1-g)}{((\epsilon g + e(1-g))((1-\epsilon)g + (1-e)(1-g)))^2} < 0.
\end{aligned}
\tag{14}
$$

Thus, as the error rates increase, z^* increases. □

2 Shunning

For Shunning, a donor is always assessed as bad when they interact with a bad recipient, and thus $P_{CB} = P_{DB} = Q_{CB} = Q_{DB} = 0$. When interacting with good individuals, we have:

$$
P_{CG} = \frac{\epsilon \hat{g}}{\epsilon \hat{g} + e(1-\hat{g})}, \qquad P_{DG} = \frac{(1-\epsilon)\hat{g}}{(1-\epsilon)\hat{g} + (1-e)(1-\hat{g})}. \tag{15}
$$

Under public assessment of reputations, $g_x = g_z = Q_{CG} g$ and $g_y = Q_{DG} g$. However, under private assessment, the probabilities of reputation changes are:

$$
\begin{aligned}
g_x^+ &= (1-g_x) Q_{CG} g, & g_x^- &= g_x((1-Q_{CG})g + 1 - g), \\
g_{x2}^+ &= (g_x - g_{x2}) Q_{CG} g, & g_{x2}^- &= g_{x2}((1-Q_{CG})g + 1 - g), \\
g_y^+ &= (1-g_y) Q_{DG} g, & g_y^- &= g_y((1-Q_{DG})g + 1 - g), \\
g_{y2}^+ &= (g_y - g_{y2}) Q_{DG} g, & g_{y2}^- &= g_{y2}((1-Q_{DG})g + 1 - g), \\
g_z^+ &= (1-g_z)(Q_{CG} g_2 + Q_{DG}(g - g_2)), & g_z^- &= g_z((1-Q_{CG})g_2 + (1-Q_{DG})(g-g_2) + 1 - g), \\
g_{z2}^+ &= (g_z - g_{z2})(Q_{CG} g_2 + Q_{DG}(g - g_2)), & g_{z2}^- &= g_{z2}((1-Q_{CG})g_2 + (1-Q_{DG})(g-g_2) + 1 - g).
\end{aligned}
\tag{16}
$$

And, at the steady state $g_i^+ = g_i^-$, we have $g_x = Q_{CG} g$, $g_y = Q_{DG} g$, $g_z = Q_{CG} g_2 + Q_{DG}(g - g_2)$, and $g_{i2} = g_i^2$.

Theorem 2.1. *Reputations converge to zero, and thus the AllD-Disc boundary is globally asymptotically stable.*

Proof. $g_x \geq g_z \geq g_y$, since $Q_{CG} \geq Q_{DG}$ and the only way in which players can be assigned as good is if they give. Discriminators cooperate at most as much as cooperators and as least as much as cheaters. Let $1 > g > 0$, then $g_x^+ - g_x^- = Q_{CG} g - g_x \leq Q_{CG} g_x - g_x = (Q_{CG} - 1) g_x < 0 \implies g_x \to 0$. Therefore, $g^* = g_x^* = g_y^* = g_z^* = 0$ at equilibrium, and Discriminators behave as defectors resulting in $\pi_z = \pi_y > \pi_x$, and thus the AllD-Disc boundary is stable. Note that this holds for both public and private assessment, since $g_x^+ - g_x$ is the same in both. Further, this holds whether or not there is bias in $\hat{g}$, since $Q_{CG} = 1$ if and only if $g = 1$. □

3 Simple Standing

For Simple Standing, a donor is always assessed as good when they interact with a bad recipient, and thus $P_{CB} = P_{DB} = Q_{CB} = Q_{DB} = 1$. When interacting with good individuals, we have:

$$P_{CG} = \frac{\epsilon\hat{g}}{\epsilon\hat{g} + e(1-\hat{g})}, \qquad P_{DG} = \frac{(1-\epsilon)\hat{g}}{(1-\epsilon)\hat{g} + (1-e)(1-\hat{g})}. \tag{17}$$

Below we consider the two cases of assessment, public and private.

3.1 Public assessment

Under public assessment of reputations, $g_x = g_z = Q_{CG}g + 1 - g$ and $g_y = Q_{DG}g + 1 - g$.

Lemma 3.1. *Assume that there is no bias ($\hat{g} = g$). $(dQ_{DG}/dg)gy + (dQ_{CG}/dg)g(1-y) - 1$ is negative if $0 \leq g < 1$ and non-negative if $g = 1$.*

Proof. If $0 \leq g < 1$, then $g = xg_x + yg_y + (1-x-y)g_z \implies 1 - y = (2 - 1/g - Q_{DG})/(Q_{CG} - Q_{DG})$ (since $Q_{CG} > Q_{DG}$), which gives us:

$$\frac{dQ_{DG}}{dg}gy + \frac{dQ_{CG}}{dg}g(1-y) - 1 = -\frac{(1-g)(e(1-e)(1-g) + (\epsilon-e)^2 g)}{((1-\epsilon)g + (1-e)(1-g))(g\epsilon + e(1-g))} < 0. \tag{18}$$

If $g = 1$, then:

$$\frac{dQ_{DG}}{dg}gy + \frac{dQ_{CG}}{dg}g(1-y) - 1 = \frac{(1-z)(\epsilon-e)^2}{\epsilon(1-\epsilon)} \geq 0, \tag{19}$$

with equality if and only if $z = 1$. □

Theorem 3.2. *The reputation dynamics converge to a unique g^*.*

Proof. Plugging in the solutions to the reputation dynamics for g_x, g_y, and g_z into $g - xg_x - yg_y + -zg_z = 0$, we obtain:

$$\begin{aligned} &\frac{(1-g)\big(c_2 g^2 + c_1 g + c_0\big)}{(\epsilon g + e(1-g))((1-\epsilon)g + (1-e)(1-g))} = 0, \\ &c_2 = (\epsilon - e)(1 - 2e + (\epsilon - e)y) > 0, \\ &c_1 = (\epsilon - 2e + 3e^2 - 2\epsilon e), \\ &c_0 = -e(1-e) < 0. \end{aligned} \tag{20}$$

which gives us $g^* = 1$ and a solution to the quadratic polynomial $p(g) = c_2 g^2 + c_1 g + c_0$ in the numerator. Note that this polynomial is concave up. Further, $p(0) = -e(1-e) < 0$ and $p(1) = y(\epsilon - e)^2 > 0$. Therefore, there must be a solution $g^* \in (0, 1)$. We may then analyze the stability of the change in reputations $\dot{g}_i = g_i^+ - g_i^-$ by linearizing

about these two equilibria. The Jacobian of this reputation system is:

$$J = \begin{pmatrix} \frac{dQ_{CG}}{dg}gx + (Q_{CG}-1)x - 1 & \frac{dQ_{CG}}{dg}gy + (Q_{CG}-1)y & \frac{dQ_{CG}}{dg}gz + (Q_{CG}-1)z \\ \frac{dQ_{DG}}{dg}gx + (Q_{DG}-1)x & \frac{dQ_{DG}}{dg}gy + (Q_{DG}-1)y - 1 & \frac{dQ_{DG}}{dg}gz + (Q_{DG}-1)z \\ \frac{dQ_{CG}}{dg}gx + (Q_{CG}-1)x & \frac{dQ_{CG}}{dg}gy + (Q_{CG}-1)y & \frac{dQ_{CG}}{dg}gz + (Q_{CG}-1)z - 1 \end{pmatrix}, \tag{21}$$

and the characteristic polynomial is:

$$(\lambda+1)^2\left(\lambda + 1 - Q_{DG}y - Q_{CG}(1-y) + 1 - \frac{dQ_{DG}}{dg}gy - \frac{dQ_{CG}}{dg}g(1-y)\right) = 0. \tag{22}$$

By Lemma 3.1, $g^* = 1 \implies \lambda_1 = \lambda_2 = -1, \lambda_3 > 0$ and the interior equilibrium $g^* \in (0,1) \implies \lambda_1 = \lambda_2 = -1, \lambda_3 < 0$. Therefore, the interior equilibrium $g^* \in (0,1)$ is the unique stable equilibrium. □

Lemma 3.3. $x^* = 0$ *at any stable equilibria.*

Proof. Since $g_x = g_z$, $\pi_x \leq \pi_z$ with equality only if $g = 1$. Further, $g = 1 \implies \pi_x = \pi_z < \pi_y$. Therefore, there cannot be any AllC players at a stable equilibrium, since AllD players, if not Discriminators, could always invade. □

Theorem 3.4. $y^* = 1$ *is stable.* $z^* = 1$ *is unstable for no bias or positive bias, and stable for negative bias if* $r > 1/(Q_{CG} - Q_{DG})$.

Proof. Define $f(z) \equiv \pi_z - \pi_y = (r(Q_{CG} - Q_{DG})z - 1)g$ and note that $y^* = 1$ and $z^* = 1$ are stable if $f(0) < 0$ and $f(1) > 0$, respectively. $g^* = 0$ cannot be a solution to the reputation dynamics, because it implies the contradictions $g_y = Q_{DG}0 + 1 - 0 = 1$ and $g_z = Q_{CG}0 + 1 - 0 = 1$. Thus, $g^* > 0 \implies f(0) = -^*g < 0$, and thus $y^* = 1$ is always stable.

Now consider the equilibrium $z^* = 1$. If there is positive bias or no bias, then $\hat{g} = (1-\lambda)g + \lambda$ for $1 > \lambda \geq 0$, and thus:

$$\begin{aligned} Q_{CG}g + 1 - 2g &= \frac{(1-g)(c_2g^2 + c_1g + c_0)}{(\epsilon\hat{g} + e(1-\hat{g}))((1-\epsilon)\hat{g} + (1-e)(1-\hat{g}))} = 0, \\ c_2 &= -(1-2e)(\epsilon - e)(1-\lambda)^2 < 0, \\ c_1 &= -(1-\lambda)(2e - 3e^2 - \epsilon + 2e\epsilon + (1 - 3e + \epsilon)(\epsilon - e)\lambda), \\ c_0 &= (e + (\epsilon - e)\lambda)(1 - \epsilon + (\epsilon - e)(1-\lambda)) > 0. \end{aligned} \tag{23}$$

The polynomial $p(g) = c_2g^2 + c_1g + c_0$ is concave down, and $p(0) = c_0 > 0$ and $p(1) = \epsilon(1-\epsilon)\lambda \geq 0$. Thus, there is no solution within $(0,1)$ leaving $g^* = 1$ as the only solution to Equation 23, which must be stable. $g^* = 1 \implies f(1) = -1$, and thus $z^* = 1$ is unstable. If there is negative bias, then $z^* = 1$ is stable if $r > 1/(Q_{CG} - Q_{DG})$. Note that g^* cannot be 1 under negative bias, since this implies that $g_z = Q_{CG} = 1$. Yet, $\hat{g} < 1 \implies P_{CG} < 1$ and $P_{DG} < 1 \implies Q_{CG} < 1$. □

Theorem 3.5. *Assume that there is no bias ($\hat{g} = g$). In addition to the stable equilibrium $y^* = 1$, the AllD-Disc boundary has either: an unstable equilibrium $(0, 1 - z_1^*, z_1^*)$ and a stable equilibrium $(0, 1 - z_2^*, z_2^*)$ with $0 < z_1^* < z_2^* < 1$; a single internal semi-stable equilibrium; or no internal equilibrium and thus $y^* = 1$ is the sole stable equilibrium.*

Proof. First we will show that g is increasing with respect to z. Define $h \equiv g_y(1 - z) + g_z z - g = Q_{DG} g(1 - z) + Q_{CG} gz + 1 - 2g = 0$. Taking dh/dz gives us:

$$k_1 \frac{dg}{dz} + k_0 = 0, \quad k_0 = (Q_{CG} - Q_{DG})g > 0, \quad k_1 = Q_{DG}(1 - z) + Q_{CG} z - 1 + \frac{dQ_{DG}}{dg} g(1 - z) + \frac{dQ_{CG}}{dg} gz - 1 < 0, \quad (24)$$

by Lemma 3.1 and $1 > Q_{CG} > Q_{DG} > 0$. Therefore, $dg/dz > 0$.

Define $f \equiv \pi_z - \pi_y = r(g_z - g_y)z - g = r(2g - 1 - Q_{DG} g) - g$ by substituting in $(g_z - g_y)z = g - g_y$. We may arrange f into a fraction with positive denominator and a cubic numerator:

$$\begin{aligned} f &= \frac{c_3 g^3 + c_2 g^2 + c_1 g + c_0}{(\epsilon g + e(1 - g))((1 - \epsilon)g + (1 - e)(1 - g))}, \\ c_3 &= -(\epsilon - e)((r - 1)(\epsilon - e) + r(1 - 2e)) < 0, \\ c_2 &= (3r - 1)(\epsilon - e)(1 - 2e) - r\epsilon(1 - \epsilon), \\ c_1 &= (2r - 1)(e^2 + \epsilon(1 - 2e)) - (3r - 1)(\epsilon - e)(1 - 2e), \\ c_0 &= -er(1 - e) < 0. \end{aligned} \quad (25)$$

The discriminant of the cubic is

$$\Delta = 18 c_3 c_2 c_1 c_0 - 4 c_2^3 c_0 + c_2^2 c_1^2 - 4 c_3 c_1^3 - 27 c_3^2 c_0^2. \quad (26)$$

Note that if $c_2 < 0$, then $r\epsilon(1 - \epsilon) > (\epsilon - e)(1 - 2e)(3r - 1) > 0$. Therefore,

$$c_1 > (2r - 1)(e^2 + \epsilon(1 - 2e)) - r(1 - \epsilon)\epsilon r(\epsilon - e)^2 + (r - 1)\epsilon(1 - 2e) + (r - 1)e^2 > 0. \quad (27)$$

Since, c_2 and c_1 cannot both be negative, there are only two sign changes in the coefficients of $f(g)$ and one sign change in the coefficients of $f(-g)$. By Descartes's rule of signs, there are either two or zero real positive roots and a sole real negative root. Since $f(g(z = 0)) < 0$ and $f(g(z = 1)) < 0$ by Theorem 3.4, the roots are either both within or both outside $[f(g(z = 0)), 1]$. If $\Delta > 0$, we have two real positive roots. If they are within $[g(z = 0), 1]$, then we have two equilibria (one must be stable and the other unstable). If they are not, then $y^* = 1$ is globally asymptotically stable. When $\Delta = 0$, we have a single real positive root of multiplicity two. If this root is within $[g(z = 0), 1]$, we have a semi-stable equilibria. Otherwise, $y^* = 1$ is globally asymptotically stable. When $\Delta < 0$, there is only one real root, which must be the negative one, and thus there is no polymorphic equilibrium on the AllD-Disc boundary. □

3.2 Private assessment

We begin by finding the equilibrium reputations. For x, y, and z, these probabilities of reputation changes are given by:

$$\begin{aligned}
g_x^+ &= (1-g_x)(Q_{CG}g+1-g), & g_x^- &= g_x(1-Q_{CG})g, \\
g_{x2}^+ &= (g_x-g_{x2})(Q_{CG}g+1-g), & g_{x2}^- &= g_{x2}(1-Q_{CG})g, \\
g_y^+ &= (1-g_y)(Q_{DG}g+1-g), & g_y^- &= g_y(1-Q_{DG})g, \\
g_{y2}^+ &= (g_y-g_{y2})(Q_{DG}g+1-g), & g_{y2}^- &= g_{y2}(1-Q_{DG})g, \\
g_z^+ &= (1-g_z)(Q_{CG}g_2+Q_{DG}(g-g_2)+1-g), & g_z^- &= g_z((1-Q_{CG})g_2+(1-Q_{DG})(g-g_2)), \\
g_{z2}^+ &= (g_z-g_{z2})(Q_{CG}g_2+Q_{DG}(g-g_2)+1-g), & g_{z2}^- &= g_{z2}((1-Q_{CG})g_2+(1-Q_{DG})(g-g_2)),
\end{aligned} \tag{28}$$

where $g_{x2}=g_x^2$, $g_{y2}=g_y^2$, $g_{z2}=g_z^2$. At any equilibrium we must have $g_i^+=g_i^-$ and so

$$g_x = Q_{CG}g+1-g, \qquad g_y = Q_{DG}g+1-g, \qquad g_z = Q_{CG}g_2+Q_{DG}(g-g_2)+1-g. \tag{29}$$

Lemma 3.6. *There is no internal strategic equilibrium $x^*, y^*, z^* > 0$ and thus no limit cycle.*

Proof. Assume there is such a point (x^*, y^*, z^*). At this point, the payoffs for each strategy are the same: $\pi_x = \pi_y \implies r(Q_{CG}-Q_{DG})gz = 1$, and $\pi_y = \pi_z \implies r(Q_{CG}-Q_{DG})g_2z = g$ (note that $g \neq 0$ or 1). Subbing the former into the latter gives us $g_2 = g^2$. However,

$$\begin{aligned}
g_2 - g^2 &= (g_x-g_y)^2x^*y^* + (g_x-g_z)^2x^*z^* + (g_y-g_z)^2y^*z^* \geq (Q_{CG}-Q_{DG})^2g^2x^*y^* \\
&= \left(\frac{(\epsilon-e)^2\hat{g}(1-\hat{g})}{(\epsilon\hat{g}+e(1-\hat{g}))((1-\epsilon)\hat{g}+(1-e)(1-\hat{g}))}\right)^2 g^2x^*y^* > 0,
\end{aligned}$$

which is a contradiction. Note that $\hat{g} \neq 0, 1$, since this would imply that $Q_{CG} = Q_{DG}$ and thus the payoffs cannot be equal. Therefore, there cannot be a limit cycle, because the imitation dynamical system is two dimensional. ☐

Theorem 3.7. *There is a unique $g^* \in (0,1)$ on the AllD-Disc boundary.*

Proof. Plugging in the solutions to the reputation dynamics for g_y and g_z into $g-(1-z)g_y+-zg_z = 0$, we obtain:

$$\begin{aligned}
&\frac{(1-g)(c_2g^2+c_1g+c_0)}{(\epsilon g+e(1-g))((1-\epsilon)g+(1-e)(1-g))} = 0, \\
&c_2 = (\epsilon-e)(1-2e+(\epsilon-e)) > 0, \\
&c_1 = -e^2(3+g_2z) - \epsilon(1+g_2z\epsilon) + 2e(1+\epsilon+g_2z\epsilon), \\
&c_0 = -e(1-e) < 0.
\end{aligned} \tag{30}$$

which gives us $g^* = 1$ and a solution to the quadratic polynomial $p(g) = c_2g^2 + c_1g + c_0$ in the numerator. Note that this polynomial is concave up. Further, $p(0) = -e(1-e) < 0$ and $p(1) = (1-zg_2)(\epsilon-e)^2 > 0$. Therefore, there must

be a solution $g^* \in (0,1)$. We may then analyze the stability of the change in reputations $\dot{g}_i = g_i^+ - g_i^-$ by linearizing about these two equilibria. The elements of the Jacobian matrix $J = \{j_{mn}\}$ of this reputation system are:

$$
\begin{aligned}
j_{11} &= \left(\frac{dQ_{DG}}{dg}g + Q_{DG} - 1\right)(1-z) - 1,\\
j_{12} &= \left(\frac{dQ_{DG}}{dg}g + Q_{DG} - 1\right)z,\\
j_{13} &= 0,\\
j_{14} &= 0,\\
j_{21} &= \left(g_2\left(\frac{dQ_{CG}}{dg} - \frac{dQ_{DG}}{dg}\right) + \frac{dQ_{DG}}{dg}g + Q_{DG} - 1\right)(1-z),\\
j_{22} &= \left(g_2\left(\frac{dQ_{CG}}{dg} - \frac{dQ_{DG}}{dg}\right) + \frac{dQ_{DG}}{dg}g + Q_{DG} - 1\right)z - 1,\\
j_{23} &= (Q_{CG} - Q_{DG})(1-z),\\
j_{24} &= (Q_{CG} - Q_{DG})z,\\
j_{31} &= gQ_{DG} + 1 - g + g_y\left(\frac{dQ_{DG}}{dg}g + Q_{DG} - 1\right)(1-z),\\
j_{32} &= g_y\left(\frac{dQ_{DG}}{dg}g + Q_{DG} - 1\right)z,\\
j_{33} &= -1,\\
j_{34} &= 0,\\
j_{41} &= g_z\left(\left(\frac{dQ_{CG}}{dg} - \frac{dQ_{DG}}{dg}\right)g_2 + \frac{dQ_{DG}}{dg}g + Q_{DG} - 1\right)(1-z),\\
j_{42} &= (Q_{CG} - Q_{DG})g_2 + Q_{DG}g + 1 - g + g_z\left(\left(\frac{dQ_{CG}}{dg} - \frac{dQ_{DG}}{dg}\right)g_2 + \frac{dQ_{DG}}{dg}g + Q_{DG} - 1\right)z,\\
j_{43} &= g_z(Q_{CG} - Q_{DG})(1-z),\\
j_{44} &= g_z(Q_{CG} - Q_{DG})z - 1,
\end{aligned}
\tag{31}
$$

and the characteristic polynomial evaluate at $g^* = 1$ is:

$$
(1+\lambda)^3\left(1 - \frac{dQ_{DG}}{dg}(1-z) - \frac{dQ_{CG}}{dg}z + \lambda\right) = 0. \tag{32}
$$

By Lemma 3.1, the eigenvalues are $\lambda_1 = \lambda_2 = \lambda_3 = -1, \lambda_4 > 0$ and thus this state is unstable and reputations cannot converge to it leaving only the interior equilibrium $g^* \in (0,1)$. □

Theorem 3.8. *Assume that there is no bias ($\hat{g} = g$). The AllC-Disc boundary is unstable. $y^* = 1$ is stable.*

Proof. On the AllC-Disc boundary with no bias ($\hat{g} = g$), $g = g_x = g_z = 1$ and thus $\pi_z = \pi_x$. $g_x \geq g_z$, since $\epsilon > e$. The only way in which players can be assigned as good is if they give, and Discriminators cooperate at most as much as

AllC players. Further, note that by substituting g_x and g_z of Equations 29 into $g = g_x(1-z) + g_z z$ gives us

$$(Q_{CG} - Q_{DG})(g - g_2)z = g(Q_{CG} - 2) + 1. \tag{33}$$

Note that $Q_{CG} - Q_{DG} \geq 0$ and $g \geq g_2 \implies g(Q_{CG} - 2) + 1 \geq 0$. $g_x^+ - g_x^- = Q_{CG}g + 1 - g - g_x \geq Q_{CG}g + 1 - 2g \geq 0$, and thus $g_x \to 1$. By $g_x = 1$, Equation 29, and $e < \frac{1}{2}$, $Q_{CG} = 1 \implies g = 1 \implies g_z = 1$. Therefore, Discriminators behave as AllC players and $\pi_z = \pi_x$. Therefore, $\pi_y - \pi_x = \pi_y - \pi_z = 1 > 0$, and the boundary is unstable. □

Theorem 3.9. *Assume that there is no bias ($\hat{g} = g$). The AllD-Disc boundary has either: an unstable equilibrium $(0, 1-z_1^*, z_1^*)$ and a stable equilibrium $(0, 1-z_2^*, z_2^*)$ with $0 < z_1^* < z_2^* < 1$; a single internal semi-stable equilibrium; or no internal equilibrium and thus $y = 1$ is globally asymptotically stable.*

Proof. Assuming that g is increasing with respect to z as in Staying under public assessment, we may then define $f \equiv \pi_z - \pi_y = r(g_z - g_y)z - g = r(2g - 1 - Q_{DG}g) - g$ by substituting in $(g_z - g_y)z = g - g_y$, and apply the arguments from Theorem 3.5. Note that f is equivalent in that Theorem and this one and thus the equilibria are determined by g. However, g maps to z differently and so the locations of these equilibria along the AllD-Disc boundary may be different. □

Theorem 3.10. *$x^* = 1$ is unstable and $y^* = 1$ is stable. Further, if the error rate is sufficiently high, $y^* = 1$ is globally asymptotically stable.*

Proof. At $z^* = 0$, $\pi_y - \pi_x = 1 > 0$. And by the above lemmas. □

4 Staying

For Staying, the probabilities that the donor is good given the observers' beliefs about their actions and the recipients' reputations with the observers are:

$$P_{CG} = \frac{\epsilon\hat{g}}{\epsilon\hat{g} + e(1-\hat{g})}, \qquad P_{DG} = \frac{(1-\epsilon)\hat{g}}{(1-\epsilon)\hat{g} + (1-e)(1-\hat{g})}. \tag{34}$$

Since observers make no assessments when the recipient is bad, we do not have P_{CB}, P_{DB}, Q_{CB}, and Q_{DB}.

4.1 Public assessment

Since a donor is not assessed when they interact with a bad recipient, $g_x = g_z = Q_{CG}$ and $g_y = Q_{DG}$.

Theorem 4.1. *The reputation dynamics converge to $g^* = 1 - y$.*

Proof. We find g^* by solving $g = xg_x + yg_y + zg_z = xQ_{CG} + yQ_{DG} + (1-x-y)Q_{CG}$ with respect to g:

$$xQ_{CG} + yQ_{DG} + (1-x-y)Q_{CG} - g = \frac{(\epsilon - e)^2 g(1-g)(g-1+y)}{(\epsilon g + (1-g)e)((1-\epsilon)ge + (1-e)(1-g))} = 0, \tag{35}$$

which gives us $g^* = 0, 1-y, 1$. We may then analyze the stability of the change in reputations $\dot{g}_i = g_i^+ - g_i^-$ by linearizing about these equilibria. The Jacobian of this reputation system is:

$$J = \begin{pmatrix} \frac{dQ_{CG}}{dg_x} - 1 & \frac{dQ_{CG}}{dg_y} & \frac{dQ_{CG}}{dg_z} \\ \frac{dQ_{DG}}{dg_x} & \frac{dQ_{DG}}{dg_y} - 1 & \frac{dQ_{DG}}{dg_z} \\ \frac{dQ_{CG}}{dg_z} & \frac{dQ_{CG}}{dg_z} & \frac{dQ_{CG}}{dg_z} - 1 \end{pmatrix}. \tag{36}$$

The eigenvalues λ_i of J at the equilibria g^* are:

$$\begin{aligned} g^* = 0 &\implies \lambda_1 = \lambda_2 = -1, \lambda_3 = \frac{(\epsilon - e)^2(1-y)}{e(1-e)} > 0, \\ g^* = 1-y &\implies \lambda_1 = \lambda_2 = -1, \lambda_3 = \frac{-(\epsilon - e)^2 y(1-y)}{(1-\epsilon)(1-y) + (1-e)y)(\epsilon(1-y) + ey)} < 0, \\ g^* = 1 &\implies \lambda_1 = \lambda_2 = -1, \lambda_3 = \frac{(\epsilon - e)^2 y}{\epsilon(1-\epsilon)} > 0. \end{aligned} \tag{37}$$

Since there are positive eigenvalues for $g^* = 0$ and $g^* = 1$, they are unstable. All of the eigenvalues for $g^* = 1-y$ are negative, and thus it is the unique stable equilibrium. Therefore, reputations will equilibrate at $g^* = 1-y$. ☐

Theorem 4.2. *$x^* = 0$ at any stable equilibria. $y^* = 1$ is stable. And, $z^* = 1$ is unstable for no bias or positive bias, and stable for negative bias if $r > 1/(Q_{CG} - Q_{DG})$.*

Proof. Since $g_x = g_z$, $\pi_x \leq \pi_z$ with equality only if $g = 1$. Further, $g = 1 \implies \pi_x = \pi_z < \pi_y$. Therefore, there cannot be any AllC players at a stable equilibrium, since AllD players, if not Discriminators, could always invade.

Define $f(z) \equiv \pi_z - \pi_y = r(Q_{CG} - Q_{DG})z - g = (r(Q_{CG} - Q_{DG}) - 1)z$, since Theorem 4.1 gives $g^* = z$. Note that $y^* = 1$ and $z^* = 1$ are stable if $f(0) < 0$ and $f(1) > 0$, respectively. $Q_{CG} - Q_{DG}$ is decreasing in y and $Q_{CG} = Q_{DG} = 0$ when $y = 1$, and thus there is a $\delta > 0$ such that $f(\delta) = 0$ and $f(z) < 0$ for all $0 < z < \delta$. Therefore, $y^* = 1$ is stable whether or not there is bias.

At equilibrium $z^* = 1$, $g^* = 1$ by Theorem 4.1 and thus $f(1) = r(Q_{CG} - Q_{DG}) - 1$. If there is no bias or positive bias, then $Q_{CG} = Q_{DG} = 1$ and thus $f(1) = -1 < 0$ and $z^* = 1$ is unstable. However, under negative bias, $1 > Q_{CG} > Q_{DG}$ even though $g^* = 1$. Therefore, $z^* = 1$ can be stable so long as $r > 1/(Q_{CG} - Q_{DG})$. ☐

Theorem 4.3. *Assume that there is no bias ($\hat{g} = g$). In addition to the stable equilibrium $y^* = 1$, the AllD-Disc boundary has either: an unstable equilibrium $(0, 1 - z_1^*, z_1^*)$ and a stable equilibrium $(0, 1 - z_2^*, z_2^*)$ with $0 < z_1^* < z_2^* < 1$; a single internal semi-stable equilibrium; or no internal equilibrium and thus $y^* = 1$ is the sole stable equilibrium.*

Proof. First we will show that g is increasing with respect to z. Define $h \equiv g_y(1-z)+g_z z-g = Q_{DG}(1-z)+Q_{CG}z-g = 0$. Taking dh/dz gives us

$$\begin{aligned} &k_1\frac{dg}{dz}+k_0=0, \quad k_0=Q_{CG}-Q_{DG}>0, \\ &k_1=\frac{dQ_{DG}}{dg}(1-z)+\frac{dQ_{CG}}{dg}z-1=\frac{-g(1-g)(\epsilon-e)^2}{((1-\epsilon)g+(1-e)(1-g))(g\epsilon+e(1-g))}<0, \end{aligned} \tag{38}$$

since $1 > Q_{CG} > Q_{DG} > 0$ and $g = (1-z)g_y + zg_z \implies z = (g - Q_{DG})/(Q_{CG} - Q_{DG})$. Therefore, $dg/dz > 0$.

Define $f \equiv \pi_z - \pi_y = r(g_z - g_y)z - g = r(g - Q_{DG}) - g$ by substituting in $(g_z - g_y)z = g - g_y$. We may arrange f into a fraction with positive denominator and a cubic numerator with the following coefficients:

$$\begin{aligned} &f=\frac{g(c_2g^2+c_1g+c_0)}{(\epsilon g+e(1-g))((1-\epsilon)g+(1-e)(1-g))} \\ &c_2=-(r-1)(\epsilon-e)^2<0, \\ &c_1=(\epsilon-e)(r(\epsilon-e)-1+2e), \\ &c_0=-e(1-e)<0. \end{aligned} \tag{39}$$

Note that $c_1 > 0$ if

$$r > \frac{1-2e}{\epsilon-e} \tag{40}$$

at which point there are two sign changes in the coefficients of $f(g)$ and no sign change in the coefficients of $f(-g)$. By Descartes's rule of signs, there are either two or zero real positive roots and zero or two real negative roots. Since $f(z=0) < 0$ and $f(z=1) < 0$ by Theorem 4.2, the roots are either both within or both outside $[f(z=0), 1]$. If the roots are within $[0,1]$, then we have two equilibria (one must be stable and the other unstable). If they are not, then $y^* = 1$ is globally asymptotically stable. When $r = (1-2e)/(\epsilon-e)$, we have a single real positive root of multiplicity two. If this root is within $[0,1]$, we have a semi-stable equilibria. Otherwise, $y^* = 1$ is globally asymptotically stable. When $r < (1-2e)/(\epsilon-e)$, there is only one real root, which must be the negative one, and thus there is no polymorphic equilibrium on the AllD-Disc boundary. By this argument and Theorem 4.2, $y^* = 1$ is globally asymptotically stable. □

4.2 Private assessment

The probabilities of reputation changes are:

$$
\begin{aligned}
g_x^+ &= (1-g_x)Q_{CG}g, & g_x^- &= g_x(1-Q_{CG})g, \\
g_{x2}^+ &= (g_x-g_{x2})Q_{CG}g, & g_{x2}^- &= g_{x2}(1-Q_{CG})g, \\
g_y^+ &= (1-g_y)Q_{DG}g, & g_y^- &= g_y(1-Q_{DG})g, \\
g_{y2}^+ &= (g_y-g_{y2})Q_{DG}g, & g_{y2}^- &= g_{y2}(1-Q_{DG})g, \\
g_z^+ &= (1-g_z)(Q_{CG}g_2+Q_{DG}(g-g_2)), & g_z^- &= g_z((1-Q_{CG})g_2+(1-Q_{DG})(g-g_2)), \\
g_{z2}^+ &= (g_z-g_{z2})(Q_{CG}g_2+Q_{DG}(g-g_2)), & g_{z2}^- &= g_{z2}((1-Q_{CG})g_2+(1-Q_{DG})(g-g_2)).
\end{aligned}
\tag{41}
$$

At the steady state $g_i^+ = g_i^-$, we have $g_{x2}g = g_x^2 g$, $g_{y2}g = g_y^2 g$, $g_{z2}g = g_z^2 g$, and

$$
g_x = Q_{CG}, \qquad g_y = Q_{DG}, \qquad g_z g = (Q_{CG}-Q_{DG})g_2 + Q_{DG}g. \tag{42}
$$

Lemma 4.4. *There is no internal equilibrium $x^*, y^*, z^* > 0$ and thus no limit cycle.*

Proof. Assume there is such a point (x^*, y^*, z^*). At this point, the payoffs for each strategy are the same: $\pi_x = \pi_y \implies r(Q_{CG}-Q_{DG})z = 1$, and $\pi_y = \pi_z \implies r(Q_{CG}-Q_{DG})g_2 z = g^2$ (note that $g \neq 0$ or $1 \implies g_x = Q_{CG}$ and $g_y = Q_{DG}$). Subbing the former into the latter gives us $g_2 = g^2$. However,

$$
\begin{aligned}
g_2 - g^2 &= (g_x-g_y)^2x^*y^* + (g_x-g_z)^2x^*z^* + (g_y-g_z)^2y^*z^* \geq (Q_{CG}-Q_{DG})^2x^*y^* \\
&= \left(\frac{(\epsilon-e)^2\hat{g}(1-\hat{g})}{(\epsilon\hat{g}+e(1-\hat{g}))((1-\epsilon)\hat{g}+(1-e)(1-\hat{g}))}\right)^2 x^*y^* > 0,
\end{aligned}
$$

which is a contradiction. Note that $\hat{g} \neq 0, 1$, since this would imply that $Q_{CG} = Q_{DG}$ and thus the payoffs cannot be equal. Therefore, there cannot be a limit cycle, because the imitation dynamical system is two dimensional. ☐

Since there are no interior equilibria to the replicator dynamics by Lemme 4.4 and $\pi_y - \pi_x = 1 > 0$ for $z = 0$, we will consider the AllC-Disc and AllD-Disc boundaries.

Theorem 4.5. *The boundary $y = 0$ and $0 < x \leq 1$ is an unstable set of equilibria.*

Proof. $g_x \geq g_z \implies g \geq g_z$, since $\epsilon > e$. The only way in which players can be assigned as good is if they give, and Discriminators cooperate at most as much as AllC players. Since $g_2 \geq g^2$,

$$
\begin{aligned}
g_z^+ - g_z^- &= (Q_{CG}-Q_{DG})g_2 + Q_{DG}g - gg_z \geq (Q_{CG}-Q_{DG})g_2 + Q_{DG}g - g^2 \\
&= \frac{g(1-g)(\epsilon-e)^2(g_2-g^2)}{(\epsilon g+e(1-g))((1-\epsilon)g+(1-e)(1-g))} \geq 0.
\end{aligned}
\tag{43}
$$

Therefore, $g_z \to 1 \implies g_x \to 1$. Discriminators behave as AllC players and thus $\pi_z = \pi_x$.

Now, linearize the joined system of replicator and reputation dynamics about $g^* = 1$ and $y^* = 0$ gives us the Jacobian matrix

$$J = \begin{pmatrix} 0 & -x & -(x-1)x(rz+x) & 0 & (x-1)x(rz+x-1) & 0 & 0 & 0 \\ 0 & 1 & 0 & 0 & 0 & 0 & 0 & 0 \\ 0 & 0 & \tau(x-1) & 0 & \tau - \tau x & 0 & 0 & 0 \\ 0 & 0 & -\frac{\tau x\left(e^2-2e\epsilon+\epsilon\right)}{(\epsilon-1)\epsilon} & -\tau & \frac{\tau(x-1)\left(e^2-2e\epsilon+\epsilon\right)}{(\epsilon-1)\epsilon} & 0 & 0 & 0 \\ 0 & 0 & \tau x & 0 & \tau(-x) & 0 & 0 & 0 \\ 0 & 0 & \tau(x+1) & 0 & \tau - \tau x & -\tau & 0 & 0 \\ 0 & 0 & -\frac{\tau x\left(e^2-2e\epsilon+\epsilon\right)}{(\epsilon-1)\epsilon} & \tau & \frac{\tau(x-1)\left(e^2-2e\epsilon+\epsilon\right)}{(\epsilon-1)\epsilon} & 0 & -\tau & 0 \\ 0 & 0 & \tau x & 0 & \tau(-(x-2)) & 0 & 0 & -\tau \end{pmatrix}, \tag{44}$$

which has eigenvalues $\lambda_j = -\tau, 0, 1$. Thus, this equilibrium of the joint replicator-reputation system is unstable. □

Theorem 4.6. *On the boundary $x = 0$ and $0 < y \leq 1$, $g = 0$ is the only solution to the reputation dynamics.*

Proof. We begin by solving for $g = xg_x + yg_y + zg_z$, which, other than $g^* = 0, 1$, gives us:

$$g^* = \frac{x + \sqrt{x^2 + 4zg_2}}{2} \in (0, 1). \tag{45}$$

On the AllD-Disc boundary, $x = 0 \implies g^* = \sqrt{zg_2}$. However, subbing this into $g_2 = xg_x^2 + (1-x)g_z^2$ and solving for g gives us:

$$g^* = 0, \tag{46}$$

$$g^* = \frac{-e(1-e)}{(1-2e)^2 e_1(1-e_1)} < 0, \tag{47}$$

$$g^* = \frac{1-2e+\epsilon-e-\sqrt{(1-2e+\epsilon-e)^2+8e(1-e)}}{4(\epsilon-e)} \leq \frac{1-2e+\epsilon-e-\sqrt{(1-2e+\epsilon-e)^2}}{4(\epsilon-e)} = 0, \tag{48}$$

$$g^* = \frac{1-2e+\epsilon-e+\sqrt{(1-2e+\epsilon-e)^2+8e(1-e)}}{4(\epsilon-e)} \geq \frac{1-2e+\epsilon-e+\sqrt{(1-2e+\epsilon-e)^2}}{4(\epsilon-e)} \tag{49}$$

$$= \frac{1-2e+\epsilon-e}{2(\epsilon-e)} = \frac{1}{2} + \frac{1-2e}{2(1-2e)(1-e_1)} = \frac{1}{2} + \frac{1}{2(1-e_1)} \geq 1, \tag{50}$$

since $\epsilon - e = (1-2e)(1-e_1)$. Therefore, the only solution is $g^* = 0$. □

Theorem 4.7. *At $z^* = 1$, any solution $g^* \in [0, 1]$ can be a solution.*

Proof. The above theorems have neglected the case where $z^* = 1$. Solving for g^* in this case gives us $g_2 = g^{*2}$, since

$$g_z^+ - g_z^- = (Q_{CG} - Q_{DG})g_2 + Q_{DG}g - g^2 = \frac{(\epsilon - e)^2 g(1-g)(g_2 - g^2)}{(\epsilon g + e(1-g))((1-\epsilon)g + (1-e)(1-g))} = 0 \tag{51}$$

$$\implies g_2 = g^{*2} \implies g_{z2}^+ - g_{z2}^- = 0. \tag{52}$$

However, all g can be solutions. The outcome at $z^* = 1$ is thus determined by the trajectory in strategy space leading to it.

□

5 Stern Judging

For Stern Judging, the probabilities that the donor is good given the observers' beliefs about their actions and the recipients' reputations with the observers are:

$$\begin{aligned} P_{CG} &= \frac{\epsilon \hat{g}}{\epsilon \hat{g} + e(1-\hat{g})}, \qquad P_{DG} = \frac{(1-\epsilon)\hat{g}}{(1-\epsilon)\hat{g} + (1-e)(1-\hat{g})}, \\ P_{CB} &= \frac{e\hat{g}}{e\hat{g} + \epsilon(1-\hat{g})}, \qquad P_{DB} = \frac{(1-e)\hat{g}}{(1-e)\hat{g} + (1-\epsilon)(1-\hat{g})}. \end{aligned} \tag{53}$$

Below we consider the two cases of assessment, public and private.

5.1 Public assessment

Under public assessment of reputations, $g_x = Q_{CG}g + Q_{CB}(1-g)$, $g_y = Q_{DG}g + Q_{DB}(1-g)$, and $g_z = Q_{CG}g + Q_{DB}(1-g)$.

Lemma 5.1. *Assume that there is no bias ($\hat{g} = g$) and define $f(g) = Q_{CB}x + Q_{DB}(1-x) - Q_{DG}y - Q_{CG}(1-y) + 1 - (dQ_{CB}/dg)(1-g)x - (dQ_{DB}/dg)(1-g)(1-x) - (dQ_{DG}/dg)gy - (dQ_{CG}/dg)g(1-y)$. Then, $f(g) > 0$ if $g \in (\frac{1}{2}, 1)$ and $f(g) \leq 0$ if $g = 1$.*

Proof. If $g \in (\frac{1}{2}, 1)$, then $g = xg_x + yg_y + (1-x-y)g_z \implies x = (Q_{CG}g(1-y) + Q_{DG}gy + Q_{DB}(1-g) - g)/((Q_{DB} - Q_{CB})(1-g))$, which gives us:

$$f(g) = \frac{(\epsilon - e)^2 g(c_4 g^4 + c_3 g^3 + c_2 g^2 + c_1 g + c_0)}{((1-e)g + (1-\epsilon)(1-g))(eg + \epsilon(1-g))((1-\epsilon)g + (1-e)(1-g))^2(\epsilon g + e(1-g))^2},$$
$$c_4 = (\epsilon - e)^2(1 - 2e + e_1(1-2e))(e_1(1-2e) + (\epsilon - e)y)) > 0,$$
$$c_3 = 2(\epsilon - e)(-e^2(y(5\epsilon + 2) - 3\epsilon + 9) + e^3(3y + 5) + e(y(\epsilon + 4)\epsilon - 3\epsilon^2 + 4) + (\epsilon - 2)\epsilon((y-1)\epsilon + 1)),$$
$$c_2 = (\epsilon - e)(e^2((9y - 10)\epsilon + 23) - 3e^3(y + 4) + e(y(-\epsilon)(\epsilon + 8) + 2y + \epsilon(3\epsilon + 7) - 11) - (\epsilon - 2)\epsilon((y-1)\epsilon + 1)),$$
$$c_1 = 2e(1-e)(\epsilon - e)(3(1-e) - y - 3\epsilon + 2y\epsilon),$$
$$c_0 = e(1-e)(e(1-e) - \epsilon(1-\epsilon)(1-y)). \tag{54}$$

The denominator is positive, leaving us only to check $p(g) = c_4 g^4 + c_3 g^3 + c_2 g^2 + c_1 g + c_0$. Since $c_4 > 0$, $p \to \infty$ as $g \to \pm\infty$. Further,

$$\begin{aligned} p(\tfrac{1}{2}) &= \frac{1}{16}(1 - \epsilon + 1 - e)((\epsilon - e)^2 y + (1-2e)^2 e_1(1 - e_1) + 4e(1-e)y) \geq 0, \\ p(1) &= \epsilon(1-\epsilon)e(1-e)y \geq 0. \end{aligned} \tag{55}$$

Taking the derivative of $p(g)$ with respect to g we find that

$$\begin{aligned} p'(g) = 2(1-2g)(\epsilon - e)(e^2 \big(g^2(-5y\epsilon - 2y + \epsilon - 5) + g(5y\epsilon + 2y - 4\epsilon + 11) - 2y\epsilon + y + 3\epsilon - 6\big) \\ + e\big(2g^2 + (g-1)g(y-3)\epsilon^2 + \epsilon(g(4(g-1)y + 2g + 1) + 2y - 3) - 5g - y + 3\big) \\ + 3e^3(g-1)(gy + g - 1)(g-1)g(\epsilon - 2)\epsilon((y-1)\epsilon + 1)). \end{aligned} \tag{56}$$

Note that $p'(\frac{1}{2}) = 0$ and $p'(1) = -4(\epsilon - e)e(1-e)y(\epsilon - \frac{1}{2}) < 0$. Therefore, $p(g)$ must be positive for $g \in [\frac{1}{2}, 1]$, and thus $f(g) > 0$ for $g \in (\frac{1}{2}, 1)$.

If $g = 1$, then $Q_{CG} = Q_{DG} = Q_{CB} = Q_{DB}$, which implies that

$$f(g) = 1 - \frac{dQ_{DG}}{dg}y - \frac{dQ_{CG}}{dg}(1-y) = \frac{-y(\epsilon - e)^2}{\epsilon(1-\epsilon)} \leq 0 \tag{57}$$

with equality if and only if $y = 0$. □

Theorem 5.2. *The reputation dynamics converge to a unique g^*.*

Proof. Plugging in the solutions to the reputation dynamics for g_x, g_y, and g_z into $g - xg_x - yg_y + -zg_z = 0$, we

obtain

$$\frac{(\epsilon-e)^2 g(1-g)\left(c_3 g^3+c_2 g^2-c_1 g+c_0\right)}{(\epsilon g+e(1-g))((1-\epsilon)g+(1-e)(1-g))(eg+\epsilon(1-g))((1-e)g+(1-\epsilon)(1-g))}=0,$$
$$\begin{aligned} c_3 &= -(\epsilon-e)(2e_1(1-2e)+(1-x+y)(\epsilon-e))<0, \\ c_2 &= (\epsilon-e)(3e(x-2)-x(\epsilon+1)+2(y-1)\epsilon-y+4), \\ c_1 &= e^2(3x-4)+e(2\epsilon+3-2x(\epsilon+1))+\epsilon(x-y\epsilon+y+\epsilon-2), \\ c_0 &= -e(1-e)(1-x)<0, \end{aligned} \tag{58}$$

which gives us $g^*=0,1$, and any solutions to the cubic polynomial $p(g)=c_3g^3+c_2g^2+c_1g+c_0$ in the numerator. Since $c_3<0$, $p(g)\to\infty$ and $p(g)\to-\infty$ as $g\to-\infty$ and $g\to\infty$, respectively. Further, $p(\frac{1}{2})=-z(\epsilon+e)(2-\epsilon-e)/8<0$ and $p(1)=e(1-e)y>0$ for $y\neq 0$. Therefore, there must be a single solution $g^*\in(\frac{1}{2},1)$ to $p(g)=0$ when $y\neq 0$. We may then analyze the stability of the change in reputations $\dot{g}_i=g_i^+-g_i^-$ by linearizing about these three equilibria. The Jacobian of the reputation system is

$$J=\begin{pmatrix} \frac{dQ_{CG}}{dg}gx+\frac{dQ_{CB}}{dg}(1-g)x+(Q_{CG}-Q_{CB})x-1 & \frac{dQ_{CG}}{dg}gy+\frac{dQ_{CB}}{dg}(1-g)y+(Q_{CG}-Q_{CB})y & \frac{dQ_{CG}}{dg}gz+\frac{dQ_{CB}}{dg}(1-g)z+(Q_{CG}-Q_{CB})z \\ \frac{dQ_{DG}}{dg}gx+\frac{dQ_{DB}}{dg}(1-g)x+(Q_{DG}-Q_{DB})x & \frac{dQ_{DG}}{dg}gy+\frac{dQ_{DB}}{dg}(1-g)y+(Q_{DG}-Q_{DB})y-1 & \frac{dQ_{DG}}{dg}gz+\frac{dQ_{DB}}{dg}(1-g)z+(Q_{DG}-Q_{DB})z \\ \frac{dQ_{CG}}{dg}gx+\frac{dQ_{DB}}{dg}(1-g)x+(Q_{CG}-Q_{DB})x & \frac{dQ_{CG}}{dg}gy+\frac{dQ_{DB}}{dg}(1-g)y+(Q_{CG}-Q_{DB})y & \frac{dQ_{CG}}{dg}gz+\frac{dQ_{DB}}{dg}(1-g)z+(Q_{CG}-Q_{DB})z-1 \end{pmatrix}, \tag{59}$$

and the characteristic polynomial is

$$(\lambda+1)^2\Big(\lambda+Q_{CB}x+Q_{DB}(1-x)-Q_{DG}y-Q_{CG}(1-y) \\ +1-\frac{dQ_{CB}}{dg}(1-g)x-\frac{dQ_{DB}}{dg}(1-g)(1-x)-\frac{dQ_{DG}}{dg}gy-\frac{dQ_{CG}}{dg}g(1-y)\Big)=0. \tag{60}$$

By Lemma 5.1, if $g^*=0,1$ and $y^*\neq 0$, then $\lambda_1=\lambda_2=-1,\lambda_3>0$ and the interior equilibrium $g^*\in(0,1)\implies\lambda_1=\lambda_2=-1,\lambda_3<0$. Therefore, the interior equilibrium $g^*\in(0,1)$ is the unique stable equilibrium. If $y^*=0$, then $g^*=1$ is the sole stable equilibrium by Lemma 5.1. □

Lemma 5.3. $x^*=0$ *at any stable equilibria.*

Proof. $\pi_x\leq\pi_z$ with equality only if $g=1$, since

$$g_z-g_x=(Q_{DB}-Q_{CB})(1-g)=\frac{g(1-g)(\epsilon-e)^2}{(1-e)g+(1-\epsilon)(1-g))(eg+\epsilon(1-g))}\geq 0. \tag{61}$$

Further, $g=0\implies\pi_x<\pi_z=\pi_y$ and $g=1\implies\pi_x=\pi_z<\pi_y$. Therefore, there cannot be any AllC players at a stable equilibrium, since AllD players, if not Discriminators, could always invade. □

Theorem 5.4. $y^*=1$ *is stable.* $z^*=1$ *is unstable for no bias or positive bias, and stable for negative bias if* $r>1/(Q_{CG}-Q_{DG})$.

Proof. Define $f(z)\equiv\pi_z-\pi_y=(r(Q_{CG}-Q_{DG})z-1)g$ and note that $y^*=1$ and $z^*=1$ are stable if $f(0)<0$ and

$f(1) > 0$, respectively. Since $g^* \in (0,1)$ by Theorem 5.2, $f(0) < 0$, and thus $y^* = 1$ is always stable.

If there is no bias or there is positive bias, then $g = 1$ when $z^* = 1 \implies f(1) = -1$, and thus $z^* = 1$ is unstable. If there is negative bias, then $z^* = 1$ is stable if $r > 1/(Q_{CG} - Q_{DG})$ (note that g cannot be 1 under negative bias, since $Q_{CG} < 1$ and $Q_{DB} < 1$). □

Theorem 5.5. *Assume that there is no bias ($\hat{g} = g$). The AllD-Disc boundary has either: an unstable equilibrium $(0, 1-z_1^*, z_1^*)$ and a stable equilibrium $(0, 1-z_2^*, z_2^*)$ with $0 < z_1^* < z_2^* < 1$; a single internal semi-stable equilibrium; or no internal equilibrium and thus $y = 1$ is globally asymptotically stable.*

Proof. First we will show that g is increasing with respect to z. Define $h \equiv g_y(1-z) + g_z z - g = Q_{DG}g(1-z) + Q_{CG}gz + Q_{DB}(1-g) - g = 0$. Taking dh/dz gives us:

$$\begin{aligned}
&k_1 \frac{dg}{dz} + k_0 = 0, \quad k_0 = (Q_{CG} - Q_{DG})g > 0, \\
&k_1 = Q_{DG}(1-z) + Q_{CG}z - Q_{DB} + \frac{dQ_{DG}}{dg}g(1-z) + \frac{dQ_{CG}}{dg}gz + \frac{dQ_{DB}}{dg}(1-g) - 1 < 0,
\end{aligned} \tag{62}$$

by Lemma 5.1 and since $Q_{CG} > Q_{DG} > 0$. Therefore, $dg/dz > 0$.

Define $f \equiv \pi_z - \pi_y = r(g_z - g_y)z - g = r(g - Q_{DG}g - Q_{DB}(1-g)) - g$ by substituting in $(g_z - g_y)z = g - g_y$. We may arrange f into a fraction with positive denominator and a quartic numerator:

$$\begin{aligned}
f &= \frac{g(c_4 g^4 + c_3 g^3 + c_2 g^2 + c_1 g + c_0)}{(\epsilon g + e(1-g))((1-\epsilon)g + (1-e)(1-g))((1-e)g + (1-\epsilon)(1-g))(eg + \epsilon(1-g))}, \\
c_4 &= (\epsilon - e)^3(1-2e)(e_1 + 2r - 1) > 0, \\
c_3 &= -(\epsilon - e)^3(1-2e)(2e_1 + 5r - 2) < 0, \\
c_2 &= (\epsilon - e)^2(1 - e(1 + 6r - e(10r - 1)) - \epsilon + 4(e + r - 2er)\epsilon - \epsilon^2), \\
c_1 &= -(\epsilon - e)^2(1 + 5e^2 r + (r-1)\epsilon - e(1 - 2\epsilon + 2r(2+\epsilon))), \\
c_0 &= -e(1-e)(r(\epsilon - e)^2 + \epsilon(1-\epsilon)) < 0.
\end{aligned} \tag{63}$$

The discriminant of the cubic is

$$\begin{aligned}
\Delta = 256c_4^3c_0^3 - 192c_4^2c_3c_1c_0^2 + 144c_4c_2c_0(c_4c_1^2 + c_3^2c_0) - 128c_4^2c_2^2c_0^2 - 80c_4c_3c_2^2c_1c_0 - 27(c_4^2c_1^4 + c_3^4c_0^2) \\
+ 18c_3c_2c_1(c_4c_1^2 + c_3^2c_0) + 16c_4c_2^4c_0 - 6c_4c_3^2c_1^2c_0 - 4(c_4c_2^3c_1^2 + c_3^3c_1^3 + c_3^2c_2^3c_0) + c_3^2c_2^2c_1^2.
\end{aligned} \tag{64}$$

Note that if $c_2 < 0$, then

$$\begin{aligned} c_1 > c_1 + c_2 &= (\epsilon - e)^2(e^2(r-1) + 2r(\epsilon - e)(1-2e) + \epsilon(r(1-2e) + 2e - \epsilon)) \\ &> (\epsilon - e)^2\epsilon(r(1-2e) + 2e - \epsilon) \\ &> (\epsilon - e)^2\epsilon(1 - 2e + 2e - \epsilon) = (\epsilon - e)^2\epsilon(1-\epsilon) > 0. \end{aligned} \tag{65}$$

Therefore, c_2 and c_1 cannot both be negative and thus there are always three sign changes in the coefficients of $f(g)$ and one sign change in the coefficients of $f(-g)$. By Descartes's rule of signs, there are either three or one real positive roots and one real negative root. There is one positive root for $g > 1$, since $f(z=1) = -1 < 0$ by Theorem 5.4 and $f \to \infty$ as $g \to \infty$. Thus there are either two or no roots within $[f(z=0), 1]$. If $\Delta > 0$, then we either have four real roots or none. However, since we always have a root greater than 1, we must have four real roots, two of which are outside of $[f(z=0), 1]$. If the other two roots are within $[g(z=0), 1]$, then we have two equilibria (one must be stable and the other unstable). If they are not, then $y^* = 1$ is globally asymptotically stable. When $\Delta = 0$, there is a real positive root of multiplicity two. If this root is within $[g(z=0), 1]$, we have a semi-stable equilibria. Otherwise, $y^* = 1$ is globally asymptotically stable. When $\Delta < 0$, there are only two real roots, one of which must be negative and the other greater than 1, which implies that there is no polymorphic equilibrium on the AllD-Disc boundary.

□

5.2 Private assessment

The probabilities of reputation changes are:

$$
\begin{aligned}
g_x^+ &= (1-g_x)(Q_{CG}g + Q_{CB}(1-g)),\\
g_x^- &= g_x((1-Q_{CG})g + (1-Q_{CB})(1-g)),\\
g_{x2}^+ &= (g_x - g_{x2})(Q_{CG}g + Q_{CB}(1-g)),\\
g_{x2}^- &= g_{x2}((1-Q_{CG})g + (1-Q_{CB})(1-g)),\\
g_y^+ &= (1-g_y)(Q_{DG}g + Q_{DB}(1-g)),\\
g_y^- &= g_y((1-Q_{DG})g + (1-Q_{DB})(1-g)),\\
g_{y2}^+ &= (g_y - g_{y2})(Q_{DG}g + Q_{DB}(1-g)),\\
g_{y2}^- &= g_{y2}((1-Q_{DG})g + (1-Q_{DB})(1-g)),\\
g_z^+ &= (1-g_z)(Q_{CG}g_2 + (Q_{DG}+Q_{CB})(g-g_2) + Q_{DB}(1-2g+g_2)),\\
g_z^- &= g_z((1-Q_{CG})g_2 + (2-Q_{DG}-Q_{CB})(g-g_2) + (1-Q_{DB})(1-2g+g_2)),\\
g_{z2}^+ &= (g_z - g_{z2})(Q_{CG}g_2 + (Q_{DG}+Q_{CB})(g-g_2) + Q_{DB}(1-2g+g_2)),\\
g_{z2}^- &= g_{z2}((1-Q_{CG})g_2 + (2-Q_{DG}-Q_{CB})(g-g_2) + (1-Q_{DB})(1-2g+g_2)).
\end{aligned}
\tag{66}
$$

Note that $1-2g+g_2$ is the probability that two Discriminators agree that a player is bad. At the steady state $g_i^+ = g_i^-$, we have $g_{x2} = g_x^2$, $g_{y2} = g_y^2$, $g_{z2} = g_z^2$, and

$$
\begin{aligned}
g_x &= Q_{CG}g + Q_{CB}(1-g),\\
g_y &= Q_{DG}g + Q_{DB}(1-g),\\
g_z &= Q_{CG}g_2 + (Q_{CB}+Q_{DG})(g-g_2) + Q_{DB}(1-2g+g_2).
\end{aligned}
\tag{67}
$$

Theorem 5.6. *Assume that there is no bias ($\hat{g} = g$). Reputations converge to $g = g_x = g_y = g_z = \frac{1}{2}$ and thus $y^* = 1$ is globally asymptotically stable.*

Proof. Note that $Q_{CG} \geq Q_{DG}$ and $Q_{DB} \geq Q_{CB}$ with equalities if and only if $g = 0$, $\frac{1}{2}$, or 1. Further,

$$
\begin{aligned}
g_x - g_y &= (Q_{CG} - Q_{DG})g + (Q_{CB} - Q_{DB})(1-g)\\
&= \frac{(2g-1)(1-g)g(\epsilon-e)^2(e(1-e) + g(1-g)(\epsilon-e)^2)}{(\epsilon g + e(1-g))((1-\epsilon)g + (1-e)(1-g))(eg + \epsilon(1-g))((1-e)g + (1-\epsilon)(1-g))},
\end{aligned}
\tag{68}
$$

which is positive if $g > \frac{1}{2}$, and negative if $g < \frac{1}{2}$. Since $g \geq g_2 \geq g^2$,

$$\begin{aligned} g_z - g_x &= (g - g_2)(Q_{DG} - Q_{CG} + Q_{CB} - Q_{DB}) + (Q_{DB} - Q_{CB})(1 - g) \\ &\geq (g - g^2)(Q_{DG} - Q_{CG} + Q_{CB} - Q_{DB}) + (Q_{DB} - Q_{CB})(1 - g) = (1 - g)(g_y - g_x) \geq 0 \text{ if } g < \tfrac{1}{2}. \end{aligned} \tag{69}$$

Further,

$$\begin{aligned} &Q_{CG}g + Q_{CB}(1 - g) - g \\ &= \frac{(\epsilon - e)^3(1 - \epsilon - e)g^2(1 - g)^2(1 - 2g)}{(\epsilon g + e(1 - g))((1 - \epsilon)g + (1 - e)(1 - g))((1 - e)g + (1 - \epsilon)(1 - g))(eg + \epsilon(1 - g))(eg + \epsilon(1 - g)}. \end{aligned} \tag{70}$$

Consider the case where $g < \frac{1}{2} \implies g_x \leq g$. Then, $g_x^+ - g_x^- = Q_{CG}g + Q_{CB}(1 - g) - g_x > Q_{CG}g + Q_{CB}(1 - g) - g > 0 \implies g_x \to \frac{1}{2} \implies g_y \to \frac{1}{2}$ and $g_z \to \frac{1}{2}$. On the other hand, consider the case where $g > \frac{1}{2}$. Then, $g_x \geq g_z \implies g_x^+ - g_x^- = Q_{CG}g + Q_{CB}(1 - g) - g_x < Q_{CG}g + Q_{CB}(1 - g) - g < 0 \implies g_x \to \frac{1}{2} \implies g_y \to \frac{1}{2}$ and $g_z \to \frac{1}{2}$. $g_z > g_x \implies g_z^+ - g_z^- = Q_{CG}g_2 + (Q_{CB} + Q_{DG})(g - g_2) + Q_{DB}(1 - 2g + g_2) - g_z < Q_{CG}g_2 + (Q_{CB} + Q_{DG})(g - g_2) + Q_{DB}(1 - 2g + g_2) - g < Q_{DB}(1 - 2g + 2g_2) + 2Q_{DG}(g - g_2) < Q_{DB} - g < 0 \implies g_z \to \frac{1}{2} \implies g_x \to \frac{1}{2}$ and $g_y \to \frac{1}{2}$. Therefore, reputations converge to $g = g_x = g_y = g_z = \frac{1}{2}$ and thus $\pi_y > \pi_z > \pi_x$. □

References

[1] Ohtsuki H, Iwasa Y. The leading eight: social norms that can maintain cooperation by indirect reciprocity. Journal of Theoretical Biology. 2006;239(4):435–444.
