# Supplementary material for: Indirect reciprocity with Bayesian reasoning and biases
Source: PLoS Comput Biol. 2024 Apr 25;20(4):e1011979. doi: 10.1371/journal.pcbi.1011979 (PMC11045068; doi:10.1371/journal.pcbi.1011979)
Supplement: S1 Fig — The benefit to cost ratio is r = 3 and the error rates are e1 = e2 = 0.01. The results are not qualitatively different from when there is no bias. (PDF) [file pcbi.1011979.s002.pdf]

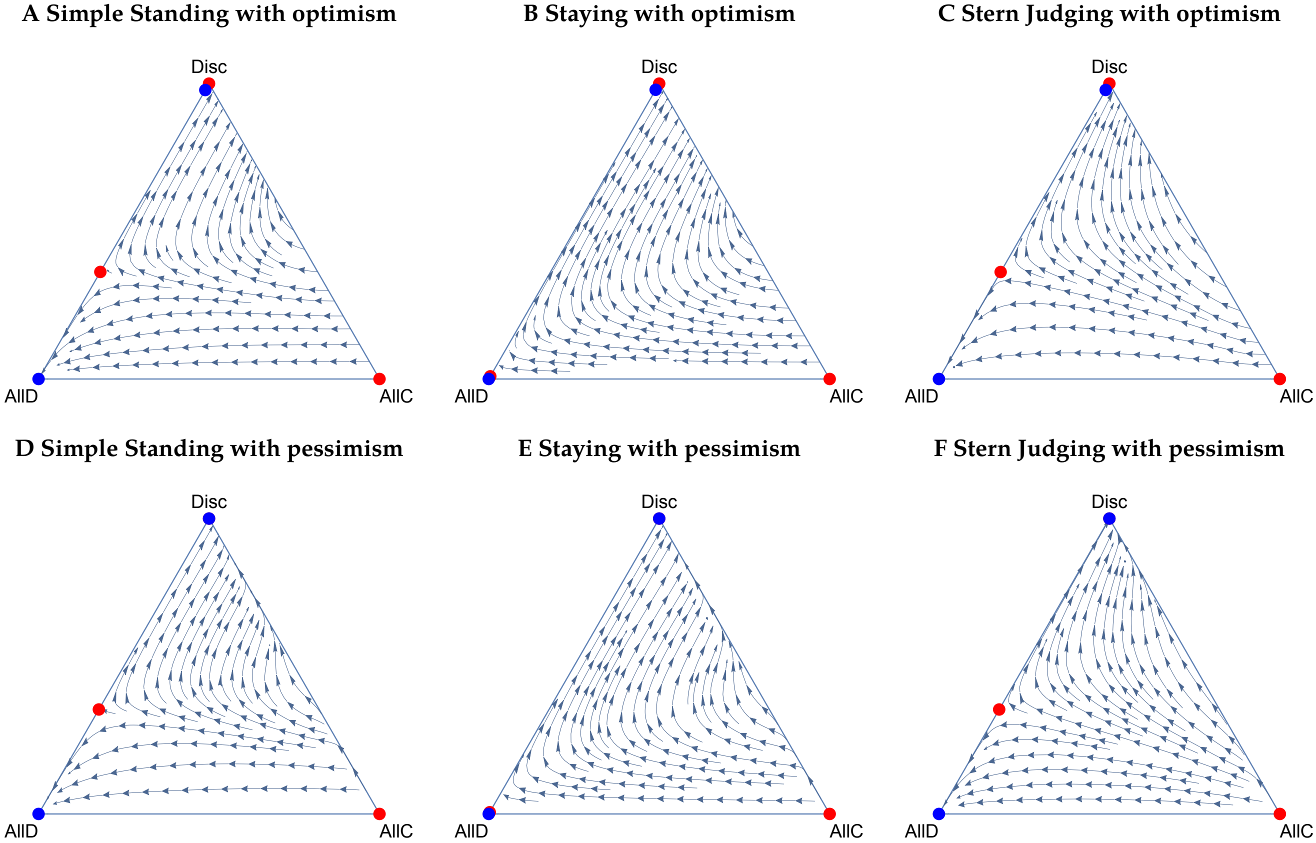
A Simple Standing with optimism
B Staying with optimism
C Stern Judging with optimism
D Simple Standing with pessimism
E Staying with pessimism
F Stern Judging with pessimism
Disc
AllD
AllC
