# Supplementary material for: Indirect reciprocity with Bayesian reasoning and biases
Source: PLoS Comput Biol. 2024 Apr 25;20(4):e1011979. doi: 10.1371/journal.pcbi.1011979 (PMC11045068; doi:10.1371/journal.pcbi.1011979)
Supplement: S2 Fig — Here AllC strategists are excluded and thus 1 − z = y. Violet curves are stable equilibria and orange curves are unstable. The results are qualitatively similar to that of Simple Standing in the main text. (PDF) [file pcbi.1011979.s003.pdf]

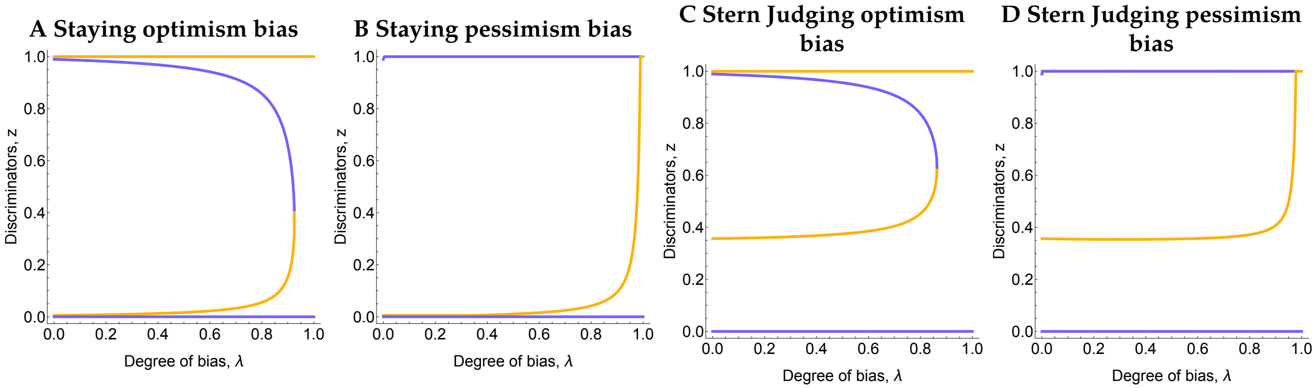
A Staying optimism bias
B Staying pessimism bias
C Stern Judging optimism bias
D Stern Judging pessimism bias
Discriminators, z
Degree of bias, λ
0.0
0.2
0.4
0.6
0.8
1.0
