# Supplementary figures and images for: Indirect reciprocity with Bayesian reasoning and biases
Source: PLoS Comput Biol. 2024 Apr 25;20(4):e1011979. doi: 10.1371/journal.pcbi.1011979 (PMC11045068; doi:10.1371/journal.pcbi.1011979)

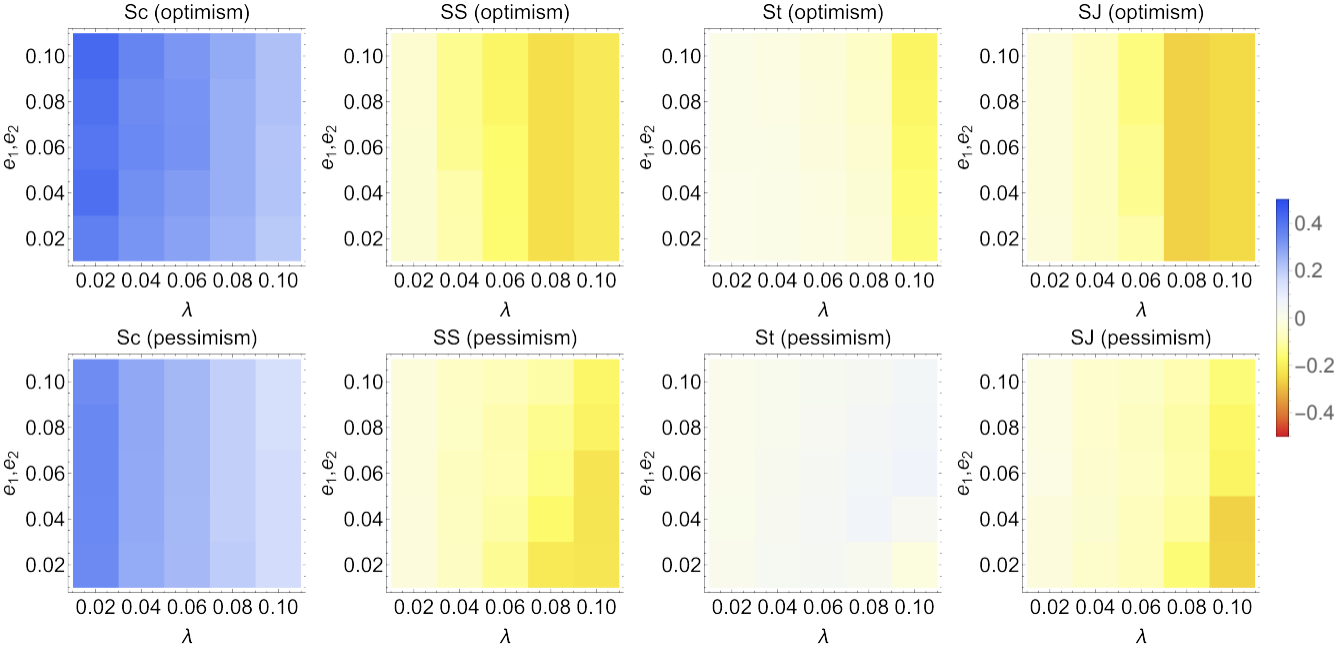

Supplement: S3 Fig — Each cell represents the average amount of cooperation under Bayesian reasoning minus the average amount of cooperation under non-reasoning. The average is over initial conditions evenly spread across the simplex and r = 3. We observe a slight synergy between the error rates and bias for Scoring under optimism bias, Simple Standing and Stern Judging under pessimism bias, and public assessment of reputations: Bayesian reasoning generally has relatively lower cooperation when biases are large and errors low. (PDF) [file pcbi.1011979.s004.pdf]

SS (optimism)

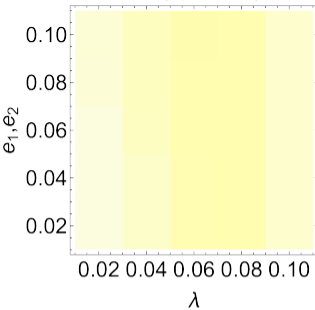

Staying (optimism)

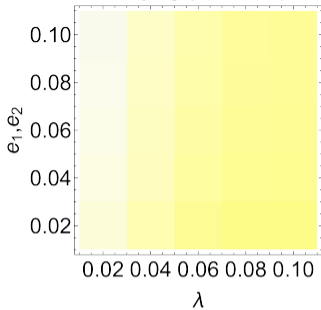

SS (pessimism)

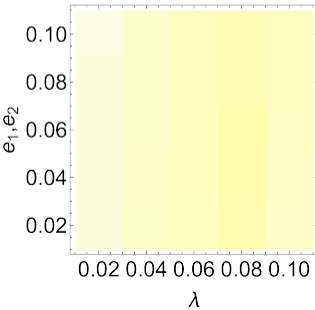

Staying (pessimism)

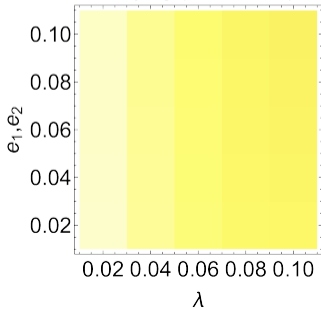

Supplement: S5 Fig — Each cell represents the average amount of cooperation under Bayesian reasoning minus the average amount of cooperation under non-reasoning. The average is over initial conditions evenly spread across the simplex and r = 3. (PDF) [file pcbi.1011979.s006.pdf]
