# Supplementary material for: Indirect reciprocity with Bayesian reasoning and biases
Source: PLoS Comput Biol. 2024 Apr 25;20(4):e1011979. doi: 10.1371/journal.pcbi.1011979 (PMC11045068; doi:10.1371/journal.pcbi.1011979)
Supplement: S4 Fig — The benefit to cost ratio is r = 3 and the error rates are e1 = e2 = 0.01. Bias has no great impact on the qualitative outcome, except that private Staying no longer has unstable equilibria on two boundaries. For optimism bias and Staying, the plot is qualitatively similar to public assessment of reputations. Negative bias results in a stable equilibrium along the AllC-Disc boundary. (PDF) [file pcbi.1011979.s005.pdf]

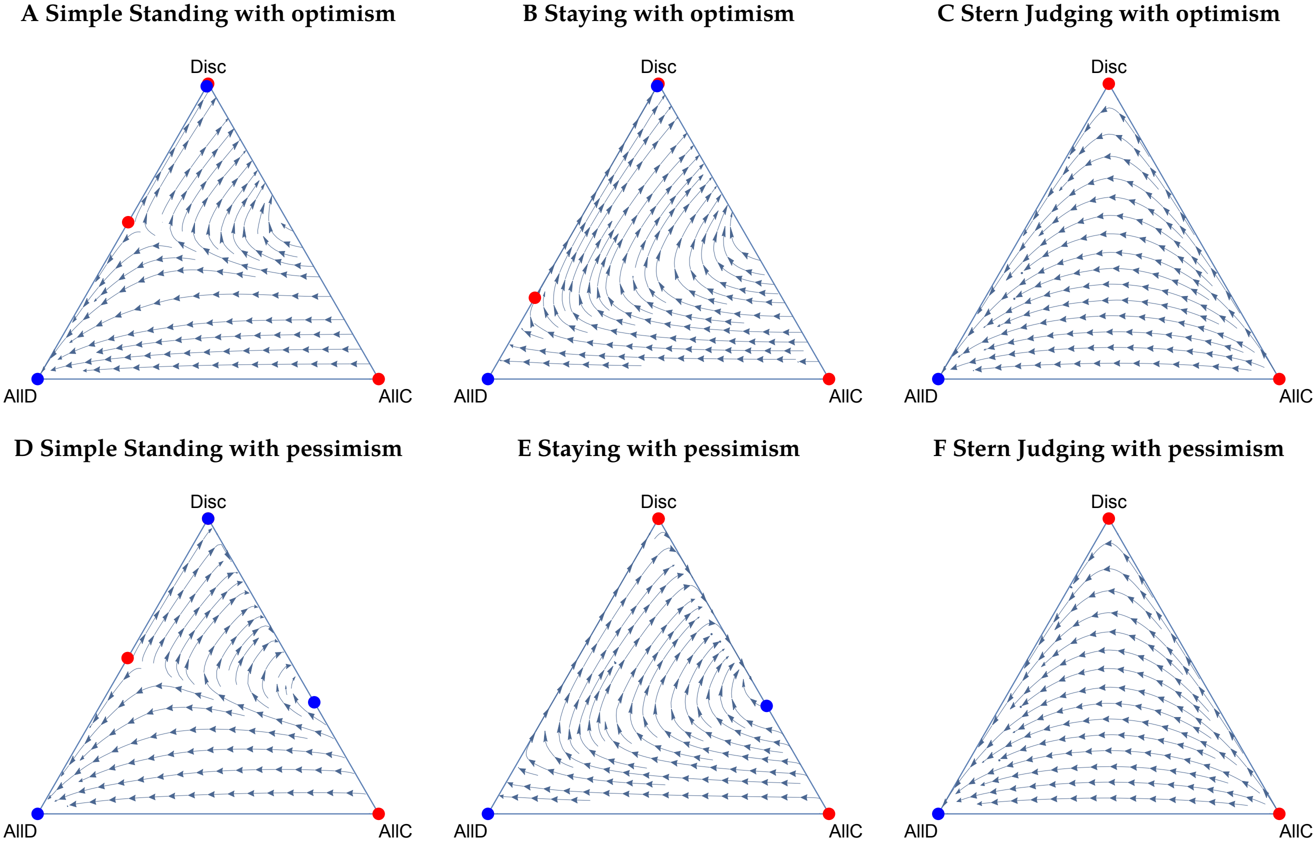

A Simple Standing with optimism
B Staying with optimism
C Stern Judging with optimism
D Simple Standing with pessimism
E Staying with pessimism
F Stern Judging with pessimism
Disc
AllD
AllC
